# *Tatdn2* is required for DNA repair to safeguard genome stability in primordial germ cells

**DOI:** 10.1093/nar/gkaf1289

**Published:** 2025-12-17

**Authors:** Hanqiao Shang, Puxuan Jiang, Zehui Wang, Zhaojun Shan, Yuze Chen, Ting Zhang, Yingshu Li, Qiang Tu

**Affiliations:** State Key Laboratory of Molecular Developmental Biology, Institute of Genetics and Developmental Biology, Chinese Academy of Sciences, 100101 Beijing, China; State Key Laboratory of Molecular Developmental Biology, Institute of Genetics and Developmental Biology, Chinese Academy of Sciences, 100101 Beijing, China; University of Chinese Academy of Sciences, 100049 Beijing, China; State Key Laboratory of Molecular Developmental Biology, Institute of Genetics and Developmental Biology, Chinese Academy of Sciences, 100101 Beijing, China; University of Chinese Academy of Sciences, 100049 Beijing, China; State Key Laboratory of Molecular Developmental Biology, Institute of Genetics and Developmental Biology, Chinese Academy of Sciences, 100101 Beijing, China; University of Chinese Academy of Sciences, 100049 Beijing, China; State Key Laboratory of Molecular Developmental Biology, Institute of Genetics and Developmental Biology, Chinese Academy of Sciences, 100101 Beijing, China; University of Chinese Academy of Sciences, 100049 Beijing, China; State Key Laboratory of Molecular Developmental Biology, Institute of Genetics and Developmental Biology, Chinese Academy of Sciences, 100101 Beijing, China; State Key Laboratory of Molecular Developmental Biology, Institute of Genetics and Developmental Biology, Chinese Academy of Sciences, 100101 Beijing, China; State Key Laboratory of Molecular Developmental Biology, Institute of Genetics and Developmental Biology, Chinese Academy of Sciences, 100101 Beijing, China; University of Chinese Academy of Sciences, 100049 Beijing, China

## Abstract

Maintaining genome integrity in germ cells is crucial for fertility and species survival. However, the DNA repair mechanisms that sustain genome integrity in primordial germ cells (PGCs), which cope with high levels of replication stress, remain largely unknown. While the TatD family of proteins, evolutionarily conserved nucleases, has been found to play roles in various DNA-related processes, their in vivo functions in vertebrates have yet to be fully elucidated. TATDN2 has recently been implicated in resolving R-loops and participating in the replication stress response in BRCA1-deficient cancer cells. Here we found that *tatdn2* exhibits conserved expression in mitotic and early meiotic germ cells across teleosts and mammals. Using medaka fish as a model, we then showed that loss of *tatdn2* leads to all-phenotypically male adults and infertility due to PGC depletion during mitotic proliferation. We further demonstrated that knockout of *tatdn2* increases R-loop accumulation and DNA damage, subsequently triggering apoptosis in PGCs. These findings indicate that *tatdn2* plays a critical role in DNA damage repair associated with R-loop resolution in mitotic PGCs. Our study provides novel insights into the physiological function of TATDN2 and the mechanisms of genome maintenance in PGCs.

## Graphical Abstract

**Graphical Abstract:**
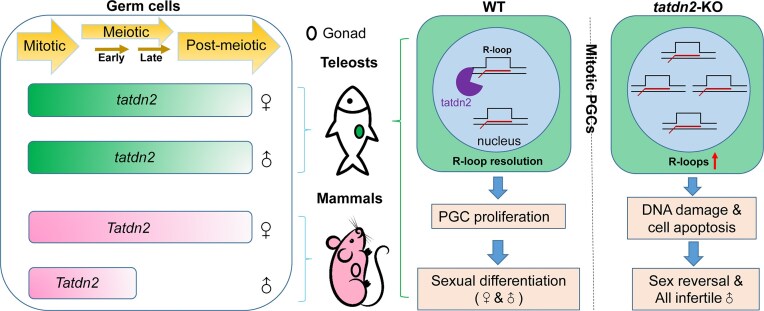

## Introduction

Germ cells must preserve their genomic integrity to ensure the transmission of intact genetic information to the next generation. This is evident by the finding that the mutation rate in gametes is nearly 100 times lower than in somatic compartments [1, 2]. The accumulation of DNA-damaging insults in germ cells can often result in a reduction or elimination of germ cells, leading to genetic aberrations, miscarriage, and infertility. After specification, primordial germ cells (PGCs) migrate to the genital ridge and proliferate rapidly, followed by sexually dimorphic meiosis. The PGC population established by mitotic proliferation is a foundation for the reproductive reserve in mammals [3]. During mitotic proliferation, PGCs encounter high levels of DNA replication stress caused by DNA base lesions, DNA interstrand crosslinks (ICLs), DNA–protein crosslinks, and transcription-replication conflicts (TRCs) [4]. Particularly, TRCs can cause accumulation of DNA–RNA hybrids (R-loops), which represent a significant source of endogenous replication stress that can compromise genome stability [5–9]. PGCs lack a G1 DNA damage-responsive cell cycle checkpoint [10] and are hypersensitive to DNA damage [11, 12]. Consequently, PGCs process unique and robust DNA repair pathways to cope with replication stress. However, the underlying mechanisms for maintaining genome stability in proliferating PGCs remain poorly understood.

Previous studies have identified a multitude of genes involved in various DNA damage repair (DDR) pathways crucial for PGC development, such as Rad54, Mcm8/Mcm9, Ercc1, Helq, and several Fanconi anemia (FA) genes [13–16]. The FA pathway, also known as the FA/BRCA pathway, is pivotal in the repair of DNA damage induced by replication stress. Dysfunction of this pathway leads to a reduction of PGCs and impaired fertility via apoptosis in mice and human patients [17]. Furthermore, the FA pathway functions are conserved across vertebrates, as evidenced by the deletion of 19 FA protein-coding genes in zebrafish also leading to PGC or GC reduction via apoptosis [18–22]. The classical role of the FA pathway is the repair of ICLs in PGCs [15, 17]. However, emerging studies have shown that the FA pathway also plays a role in resolving TRC-induced R-loops in PGCs [23–26].

TatD enzymes are conserved proteins present in organisms across all three kingdoms of life. The prototype *Escherichia coli* TatD has been characterized as a 3′–5′ exonuclease with activity on single-stranded DNA and RNA substrates, implicated in oxidative DNA damage repair [27]. Subsequently, TatD homologs have been associated with various DNA-related processes, including DNA repair and apoptosis, in lower organisms [28, 29], and parasite virulence in human protozoan parasites [30–33]. The functions of TatD proteins in vertebrates, which possess three homologs, are largely unknown, except that knockdown of zebrafish *tatdn1* leads to delays in cell cycle progression and abnormal chromosomal segregation [34]. Recently, human TATDN1 and TATDN3, together with *E. coli* TatD, have been found to possess apurinic/apyrimidinic (AP) endonuclease activity, in addition to exonuclease activity [35]. Meanwhile, human TATDN2 possesses RNA 3′ exonuclease and endonuclease activity to resolve R-loops and participates in response to replication stress in BRCA1-deficient cancer cells [36]. However, the biological significance of TATDN2 under physiological conditions remains unknown.

The medaka fish (*Oryzias latipes*) is a well-established model organism with a male heterogametic sex-determining system (the XX–XY system), similar to mammals [37]. In medaka, PGCs divide once or twice during their migratory period before arriving at the gonadal primordium at stage 33. Following this, PGCs undergo sexually dimorphic proliferation, with the sex-determining gene *dmy* (DM-domain gene on the Y chromosome) beginning to be expressed in male gonadal somatic cells [38, 39]. Since germ cells are important for sex differentiation, a reduction in PGCs in medaka impairs fertility and leads to female-to-male sex reversal [40]. These characteristics make medaka an ideal model for studying germ cell development.

In this study, we utilized medaka fish to investigate the in vivo functions of *tatdn2*. Our data showed that *tatdn2* is specifically expressed in germ cells and broadly across all their developmental stages after specification. Functional experiments demonstrated that *tatdn2* deficiency results in an all-phenotypically male population and infertility due to PGC depletion during proliferation. We further revealed that knockout of *tatdn2* increases DNA damage and R-loop accumulation and subsequently causes apoptosis of PGCs. Collectively, our findings suggest that *tatdn2* is essential for the cellular response to R-loop-related DNA damage in mitotic PGCs.

## Materials and methods

### Fish stocks and ethics

The homemade VGQ strain was selected from the offspring of Hd-rR-Tg(*vasa:EGFP*) and Qurt crossing. It exhibits both sexually dimorphic pigmentation and germ-cell-specific expression of EGFP [41]. All fish and embryos were raised and staged according to established protocols [42]. The animal work in this study was conducted in accordance with protocols approved by the Institutional Animal Care and Use Committee at the Institute of Genetics and Developmental Biology, Chinese Academy of Sciences.

### RNA extraction and qRT-PCR

Fry were separated into head and trunk regions. The head part was used for genotyping, and the trunk region containing the gonad was used for quantitative reverse transcription polymerase chain reaction (qRT-PCR) detection. Total RNA was extracted using Trizol (15596026CN, Invitrogen) following the manufacturer’s protocol. After DNase treatment, 1 µg of total RNA was used to synthesize complementary DNA using random hexamers and the Superscript III First-Strand Synthesis System for RT-PCR (18080051, Invitrogen). Real-time PCR was performed using iTaq Universal SYBR Green Supermix (1725121, Bio-Rad) on a CFX Opus Real-Time PCR System (Bio-Rad). The relative amounts of messenger RNAs (mRNAs) were calculated using the $\Delta$$\Delta$ Ct method. Primers are listed in Supplementary Table S1.

### *In situ* hybridization

Chromogenic whole-mount *in situ* hybridization was performed as previously described with slight modifications [43]. DIG-labeled RNA probes were transcribed from PCR products using a DIG labeling kit (11277073910, Roche). Primers are listed in Supplementary Table S1. The hybridized probes were detected with an antibody against digoxigenin (DIG) conjugated to alkaline phosphatase (11093274910, Roche, 1:3000).

Fluorescent ISH was performed using a hybridization chain reaction (HCR) [44]. Embryos were dechorionated using tweezers and fixed in 4% paraformaldehyde (PFA). Probes, buffers, and hairpins for third-generation *in situ* hybridization chain reaction (HCR) experiments were obtained from Molecular Instruments. Following HCR, larvae were immunostained with anti-EGFP and anti-DsRed antibodies.

### Whole mount immunofluorescence

Dechorionated embryos were fixed in 4% PFA at 4°C. The larvae were permeabilized with 20 µg/ml proteinase K for 45 min at room temperature. After blocking with 20% fetal bovine serum for 1 h at room temperature, the embryos were incubated with the indicated primary antibodies diluted in blocking solution overnight at 4°C. Microtubules were stained with mouse α-tubulin antibody (Sigma–Aldrich, T9026, 1:200) to label the cytoskeleton. Primary antibodies of DDR proteins that have been previously used in fish models were rabbit anti- $\gamma$ H2AX (GTX127342, GeneTex, 1:250) and rabbit anti-Parp1 (Abcam, ab194586, 1:200) [45].

S9.6 staining was performed as previously described, with minor modifications [46]. Fixed embryos were de-skinned and then refixed in 100% methanol at −20°C. After permeabilization in 1% Triton X-100 for 1 h, larvae were treated with the indicated RNase sets in a staining buffer supplemented with 3 mM MgCl$_{2}$ for 2 h at 37°C prior to blocking. The RNases and their working concentrations were as follows: RNase III (M0245S, NEB, 10 U/ml), RNase T1 (EN0541, ThermoFisher, 5 U/µl), and RNase H (M0297S, NEB, 100 U/ml). Larvae were then incubated with S9.6 antibody (ENH001, Kerafast, 1:200). The broad-spectrum RNase A was not used as a control due to its significant and difficult-to-control RNase H-like activity, a known confounding factor in R-loop detection, including immunofluorescence-based assays in mammalian cells [46–48]. Consistent with this, we observed that RNase A treatment eliminated the nuclear S9.6 signal in medaka PGCs even under high-salt conditions (500 mM NaCl) (Supplementary Fig. S1).

Chicken anti-EGFP (ab13970, Abcam, 1:500) and/or rabbit anti-DsRed (632496, Clontech, 1:500) were used to recover the signals of EGFP and/or DsRed. After washing, embryos were then incubated with secondary antibodies overnight at 4°C. Secondary antibodies used were Alexa Fluor 488-conjugated goat anti-chicken IgY (ab150173, Abcam, 1:300), Alexa Fluor 568-conjugated goat anti-rabbit IgG (ab175696, Abcam, 1:300), and Alexa Fluor 647-conjugated goat anti-mouse IgG (ab150119, Abcam, 1:300). Stained embryos were counterstained with DAPI and embedded in SlowFade Gold Antifade Reagents (S36936, Molecular Probes).

### Generation of stable mutant fish lines

Two crRNAs targeting exon 3 (5′-gcactgctaccatccccagg-3′) and exon 4 (5′-cctgatcttactgccgacct-3′) of *tatdn2* were utilized. Cas9 mRNA and sgRNA were injected into one-cell-stage embryos of the VGQ strain. Injected embryos were raised to adulthood and outcrossed with wild-type fish to identify germline mutations. Heterozygous fish were intercrossed to produce homozygous individuals for histological and phenotyping analyses. The mutation was introduced into the *vasa:EGFP/sox9b:dsRed* transgenic background by outcrossing *tatdn2* heterozygotes.

### Genotyping and phenotyping

Unless specifically stated otherwise, dechorionated embryos or hatched fry were dissected into the tail part for genotyping and the trunk region for phenotyping or biological molecular detection. Genomic DNAs from the caudal fin of adult fish or the tail part of embryos were obtained by alkaline lysis. All PCR primers are listed in Supplementary Table S1. For phenotyping, fish were anesthetized before body length and weight were measured using a ruler and analytical balance, respectively. Then, the fish were photographed for gross morphology with a digital camera (EOS700D, Canon). Other morphological observations were conducted under a dissecting microscope (M205FA, Leica). Fertility testing of *tatdn2* mutants was done by naturally pairing mutant males with wild-type females, as the mutants were phenotypically all male.

Genotypic sex (XY/XX) was determined by the *dmy* gene. Phenotypic sex (male/female) was assessed based on gonadal histology and secondary sex characteristics, indicated by the shapes of the anal and dorsal fins and urinogenital papillae. Phenotypic males displayed sharp, long anal fins and deeply cut dorsal fins, while phenotypic females had round, short anal fins, uncut dorsal fins, and obvious urinogenital papillae.

### Hematoxylin and eosin (H&E) staining

Dissected gonads were fixed in Bouin’s fluid (HT10132, Sigma–Aldrich), embedded in paraffin, and sectioned at a thickness of 5 µm. Sections were deparaffinized in xylene and rehydrated in gradient alcohols, incubated in hematoxylin solution, rinsed in tap water to remove excess hematoxylin, differentiated with 1% acid alcohol, and then rinsed in tap water. Finally, sections were stained with eosin, dehydrated in gradient ethanol and xylene, and mounted with Eukitt (03989, Sigma–Aldrich).

### Detection of cell apoptosis

Click-iT Alexa Fluor 647 nm TUNEL kit (C10247, ThermoFisher) was used to detect EdU according to the manufacturer’s instructions. Larvae were then immunostained against EGFP to label the PGCs, followed by EdU detection. To assess nuclear fragmentation, the microtubular structure of the cytoskeleton was immunostained with mouse anti-$\alpha$ -tubulin antibody (T9026, Sigma–Aldrich, 1:200) to facilitate the observation of cell shape, in addition to immunostaining against EGFP. The stained embryos were then examined with DAPI.

### Confocal imaging and image processing

Confocal images were obtained using a confocal microscope (LSM780 or LSM980, ZEISS). For live imaging, embryos were mounted in 0.6% low-melting-point agarose and imaged using a water Plan-Apochromat 40× objective. For PFA-fixed samples, objectives of 40× dry, 63×, or 100× oil were used. Fiji was used to adjust the contrast to highlight biological relevance and to add a scale bar. In all cases, images of control and experimental embryos were adjusted similarly.

### Sequence, structure, and transcriptomic analysis

All genomic data were obtained from the ENSEMBL or NCBI genome database. Sequence alignment was performed using ClustalW algorithm. The predicted structures of TATDN2 were from AlphaFold3 (https://alphafold.ebi.ac.uk/). Icons of species were downloaded from PhyloPic (https://www.phylopic.org). Bulk RNA-seq data of teleosts *tatdn2* was extracted from FEVER (https://fever.sk8.inrae.fr/) [49]. Bulk RNA-seq data of mammals *TATDN2* was retrieved from Evo-devo mammalian organs (https://apps.kaessmannlab.org/evodevoapp/) [50]. The processed single-cell RNA sequencing (scRNA-seq) data from GSE191137 (zebrafish ovary) [51], CRA003925 (zebrafish testis) [52], GSE149629 (macaque ovary) [53], E-MTAB-8979 (macaque testis) [54], GSE128553 (mouse ovary) [55], GSE109033 (mouse testis) [56], and GSE106487 (human testis) [57] were retrieved from NCBI-GEO, EMBL-EBI, or BIG-NGDC. ScRNA-seq datasets of medaka gonad were generated by our lab and will be published elsewhere. Data preprocessing and clustering were performed using Seurat v4.3.0, following the previously published analysis parameters. Plots were generated using R/ggplot2 from scaled and normalized datasets.

### Quantification and statistical analysis

Statistical analyses were performed from the results of three independent experiments. Bar plots were presented as mean ± SEM or SD. Experimental differences were tested for significance with a two-tailed unpaired t-test. “Ns” indicates no significant differences. A *P*-value <.05 was considered significant, denoted as *P < .05, **P < .01, ***P < .001, ****P < .0001.

## Results

### *Tatdn2* exhibits germ cell-specific expression across mitotic, meiotic, and post-meiotic stages

In a previous study, we developed an effective multiplex RNA sequencing approach named Decode-seq, which identified multiple genes with sexually dimorphic expression in medaka gonads [41]. *Tatdn2*, a previously uncharacterized gene until recently [36], emerged as a differentially expressed candidate in this analysis. TATDN2 appears evolutionarily conserved across metazoans based on sequences from GenBank. Structural predictions revealed that TATDN2 proteins contain an N-terminal disordered region and a C-terminal TatD nuclease domain [35]. Sequence and structural alignments demonstrated high conservation of the TatD domain between medaka and human TATDN2 (Supplementary Figs S2 and S3), suggesting conserved biochemical functions.

**Figure 1.**
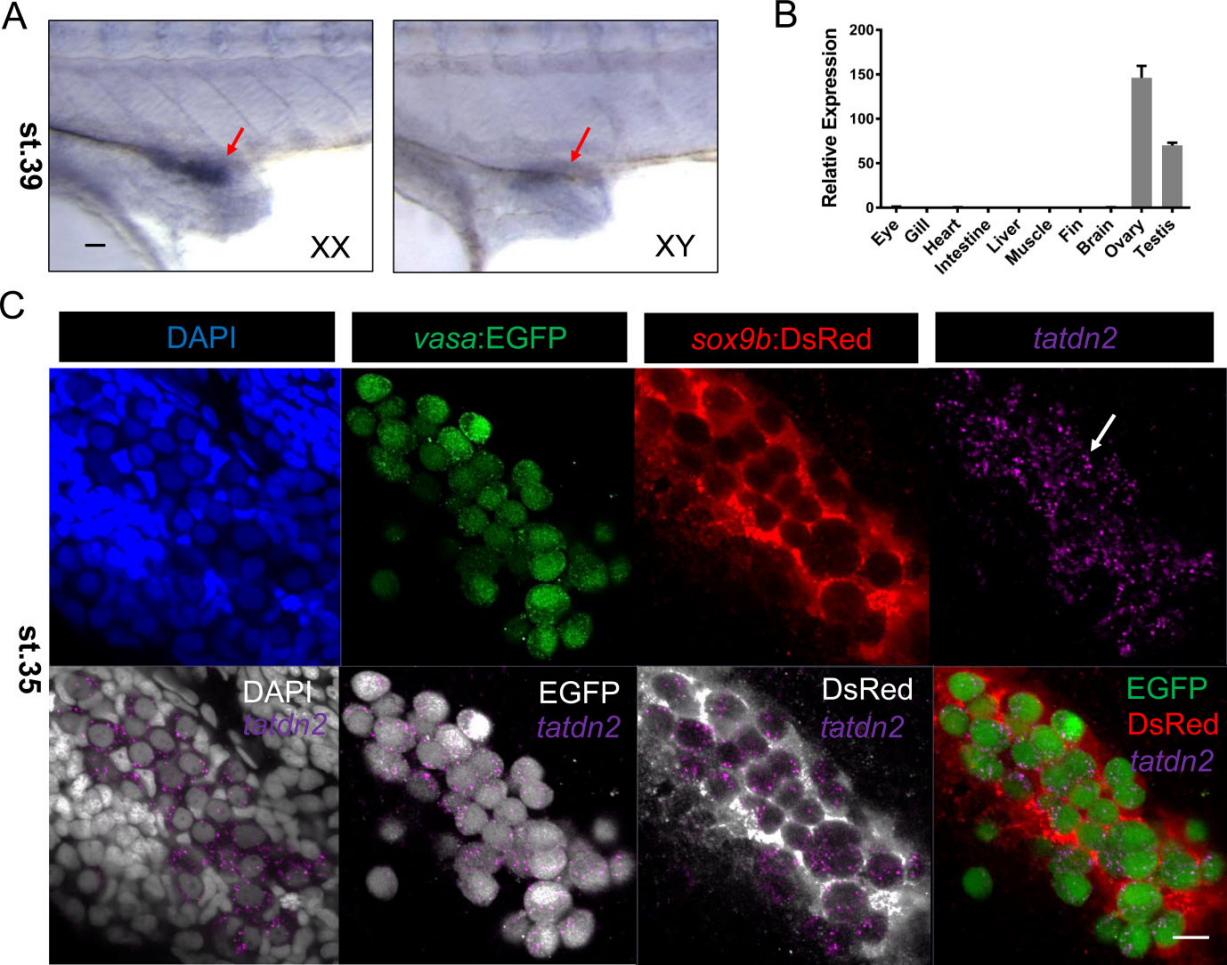
Germ cell-specific expression of *tatdn2* in medaka. **(A)** Spatial expression pattern of *tatdn2* mRNA at stage 39 by whole mount *in situ* hybridization (WISH). Arrowheads indicate the specific signal for *tatdn2* transcripts in the gonad primordium of both male and female embryos. Scale bar = 20 µm. **(B)** Tissue distribution pattern of *tatdn2* in adult medaka by qRT-PCR, showing predominant expression in both ovary and testis. **(C)** Fluorescent *in situ* hybridization of *tatdn2* mRNA on embryos at stage 35. Immunofluorescence staining for EGFP and DsRed was performed after HCR. *tatdn2* transcripts (purple) were preferentially localized in the EGFP-labeled germ cells rather than DsRed-labeled somatic cells. Note that EGFP and DsRed are distributed in both nucleus and cytoplasm. Scale bar = 10 µm.

Whole-mount in situ hybridization at stage 39 showed abundant *tatdn2* transcripts in the gonadal primordium of both sexes in medaka (Fig. 1A and Supplementary Fig. S4A). qRT-PCR confirmed predominant gonadal expression in adults (Fig. 1B), a pattern conserved across 12 teleost species according to transcriptomic data (Supplementary Fig. S4B). Together, these findings demonstrate that *tatdn2* is specifically expressed in both embryonic and adult gonads.

To determine whether *tatdn2* is expressed in germ cells or gonadal somatic cells, we performed fluorescence *in situ* hybridization using a double transgenic fish line of which the germline and soma are labeled by *vasa:EGFP* and *sox9b:dsRed*,respectively [58]. At stage 35 (initiation of PGC proliferation after specification and migration), *tatdn2* signals specifically localized to EGFP-labeled germ cells rather than DsRed-labeled somatic cells (Fig. 1C). This germline-restricted expression persisted through stage 39, when gonadal sex differentiation begins (data not shown). Furthermore, scRNA-seq of differentiated medaka gonads (manuscript in preparation) revealed *tatdn2* expression in all germ cell types (mitotic, meiotic, and post-meiotic) but not in somatic cells (Supplementary Fig. S5A and B). Cross-species analysis in zebrafish showed identical expression patterns (Supplementary Fig. S5C and D) [51, 52]. Moreover, *tatdn2* transcripts were maternally inherited, indicating their expression in late oocytes (Supplementary Fig. S4C and D).

These findings establish that medaka *tatdn2* exhibits germ-cell-specific expression throughout mitotic, meiotic, and post-meiotic developmental stages after specification.

### *TATDN2* exhibits conserved and divergent expression patterns in mammals

**Figure 2.**
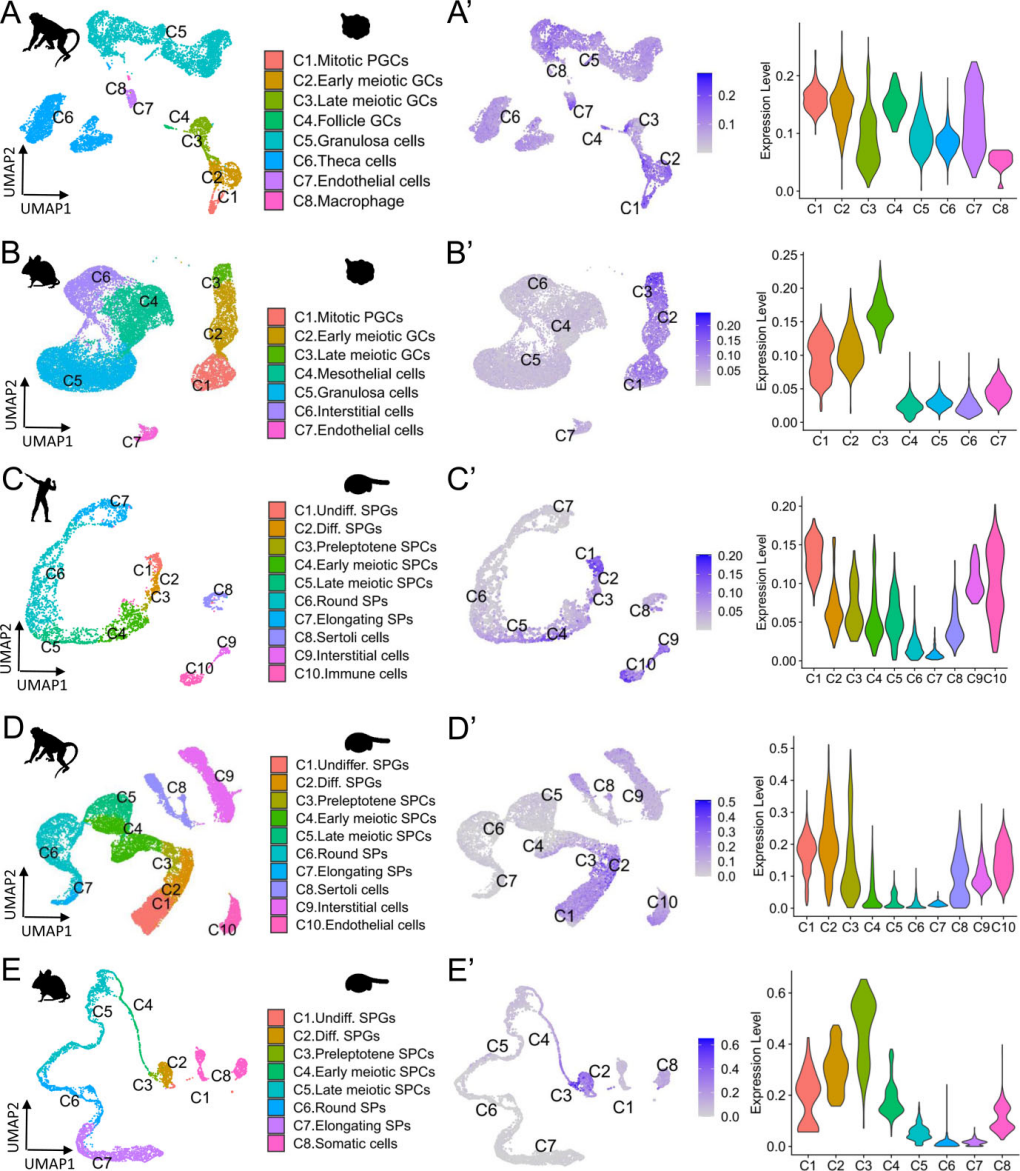
Conserved expression of *Tatdn2* in mitotic and early meiotic germ cells across teleosts and mammals. (**A**–**E**) UMAP plots showing cell identities in embryonic ovary (macaque and mouse) and adult testis (human, macaque, and mouse) based on single-cell RNA sequencing data. Each cell type is labeled with a distinct color and number. (**A’**–**E’**) UMAP plots (right panel) and violin plots (left panel) of *Tatdn2* expression across all cell types. In embryonic ovary, *Tatdn2* is highly expressed in mitotic PGCs, meiotic germ cells, and oogenesis stage FGCs. In adult testes, *Tatdn2* is predominantly expressed in spermatogonia (SPGs) with proliferating activity, preleptotene spermatocytes (SPCs) undergoing DNA replication before meiotic entry, and early meiotic SPCs.

We examined the expression pattern of *TATDN2* in mammals to determine if it is conserved across species. First, we analyzed the tissue distribution of *TATDN2* transcripts in mammals. According to multiple data resources [50, 59], *TATDN2* is broadly expressed in adult tissues, including the ovary and testis. We then analyzed the cellular expression pattern in the gonad based on previously published scRNA-seq data [53–57]. In the ovary from macaque and mouse, *TATDN2* is widely expressed in all germ cell types detected at these stages, including mitotic PGCs, meiotic germ cells, and oogenesis stage FGCs (Fig. 2A and B). In the testis from human, macaque, and mouse, as shown in Fig. 2C–E, *TATDN2* is highly expressed in spermatogonia (SPGs) with proliferating activity, preleptotene spermatocytes (SPCs), which undergo DNA replication before meiotic entry, and early meiotic SPCs, but not expressed or at a low level in late meiotic SPCs and spermatids (SPs). However, human late meiotic SPCs showed persistent *TATDN2* expression (Fig. 2C), indicating species-specific divergence. In addition, there was scattered expression of *TATDN2* in several cell subtypes of both ovarian and testicular somatic cells.

Collectively, these results of cross-species analysis show that *TATDN2* exhibits a conserved expression pattern in mitotic germ cells across teleosts and mammals, suggesting its conserved and essential role in these cells.

### Genetic ablation of *tatdn2* leads to all-phenotypically male adults and infertility

**Figure 3.**
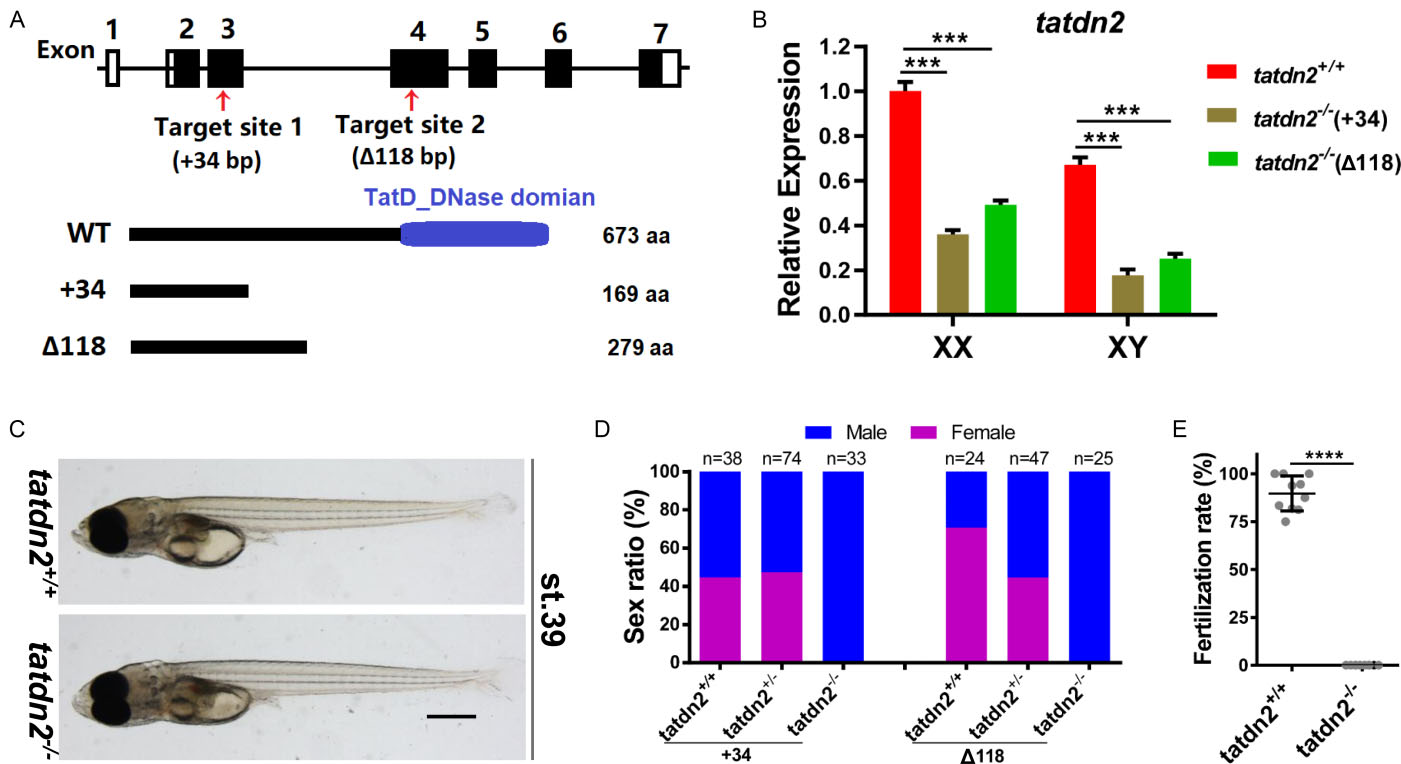
Knockout of *tatdn2* results in an all-phenotypically male population and infertility. **(A)** Schematic of the *tatdn2* genomic locus, showing wild-type and mutant protein structures. Exons are represented by dark boxes, and gRNA target sites are indicated by red arrows. Two independent mutant lines, tatdn2 +34 and tatdn2 $\Delta$118, were generated. **(B)** qRT-PCR analysis of *tatdn2* expression in stage 35 mutant embryos. **(C)** Morphology of *tatdn2* mutant embryos at stage 39 (lateral view). Offspring from heterozygous intercrosses were photographed and genotyped. Scale bar = 500 µm. **(D)** Genotype distribution and sex ratio of adult *tatdn2* mutants. **(E)** Fertility assessment of adult *tatdn2* mutants at ~120 dpf. Wild-type males were used as controls.

To investigate the *in vivo* functions of *tatdn2* at the organism level, we created stable mutant medaka using CRISPR/Cas9 technology. Two guide RNAs were designed to target the third and fourth exons of the *tatdn2* locus. This resulted in the generation of two distinct mutant alleles: one with a 34 bp insertion in exon 3, and the other with a 121 bp deletion and 3 bp insertion in exon 4. We denoted these two alleles as “+34” and “$\Delta$118,” respectively. Both mutant alleles introduce frameshift mutations, leading to premature truncation upstream of the predicted TatD nuclease domain (Fig. 3A). qRT-PCR at stage 35 revealed a significant reduction in *tatdn2* mRNA levels (Fig. 3B), suggesting nonsense-mediated decay and minimal mutant protein production. As both alleles showed identical phenotypes, the $\Delta$118 mutant was used for detailed analyses.

Morphological observation of the fry offspring at stage 39 from *tatdn2* heterozygous intercrosses showed that zygotic loss of *tatdn2* does not affect embryonic development (Fig. 3C). We then investigated the phenotype of F2 homozygotes at the adult stage. We found that all of the mutant adults are phenotypically male at ~120 days post-fertilization (dpf) (Fig. 3D). Fertility testing reveals that the all-male mutants are able to induce female spawning but fail to fertilize the eggs after natural mating with wild-type females (Fig. 3E). In addition, our data showed that the mutations are inherited in expected Mendelian ratios (1:2:1) of homozygous to heterozygous to wild-type (Fig. 3D). This indicates that the survival of homozygous mutant fish is unaffected. Consistently, the mutant adults display normal gross morphology and visceral organs, except for the gonad, with unaffected body length and body weight, compared to wild-type males (Supplementary Fig. S6).

We conclude that the knockout of *tatdn2* does not affect embryonic development or somatic growth but leads to all-phenotypically male adults and infertility.

### Knockout of *tatdn2* leads to female-to-male sex reversal and azoospermia

**Figure 4.**
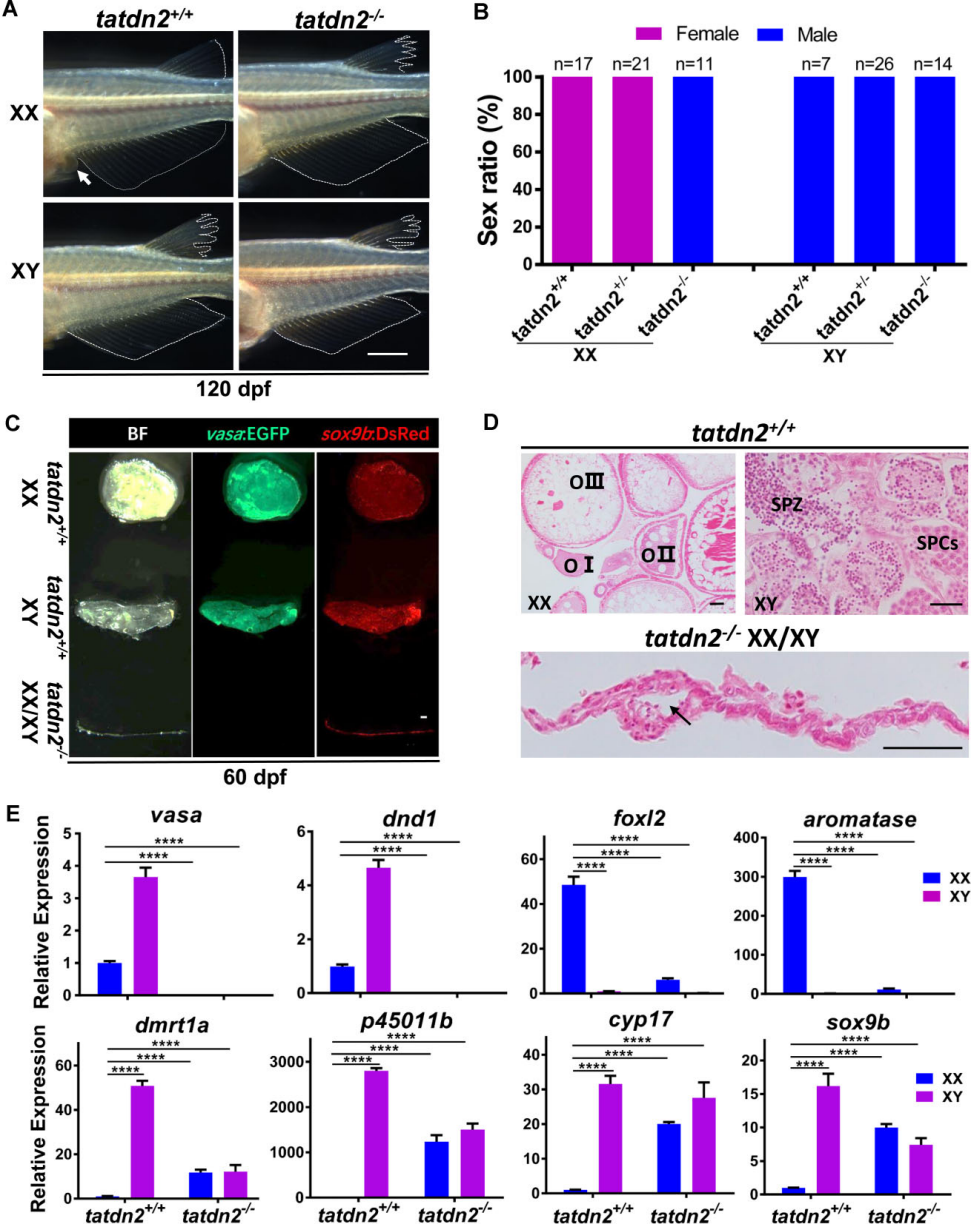
Knockout of *tatdn2* results in female-to-male sex reversal and azoospermia. **(A)** Secondary sex characteristics of adult *tatdn2*-null fish at ~120 dpf. The anal fin and dorsal fin are outlined by a dotted line, and the urinogenital papillae are indicated by an arrow. Anterior is to the left. Scale bar = 2 mm. **(B)** Genotypic and phenotypic sex distribution of *tatdn2* mutants. **(C)** Morphology of dissected gonads of *tatdn2* mutants at ~60 dpf. Bright-field (BF). Scale bar = 100 µm. Representative tube-like gonads from both XX and XY mutants are shown. **(D)** Histological sections of gonads from *tatdn2* mutants by H&E staining. The thin tube-like gonad was sectioned longitudinally. Arrow indicates the lumen structure. Oocytes (OI–III), spermatocytes (SPCs), and spermatozoa (SPZ) are labeled. Scale bar = 200 µm. **(E)** Expression profile of gonad marker genes at ~60 dpf by qRT-PCR. Germ cell markers: *vasa* and *dnd1*; female-specific markers: *foxl2* and *aromatase*; male-specific markers: *dmrt1a, p45011b, cyp17*, and *sox9b*. Note that some data points exceed the *Y*-axis limits.

To gain a better understanding of the gonad-specific defects in *tatdn2* mutants, we conducted a thorough phenotypic analysis. First, we determined the genotypic and phenotypic sex of the *tatdn2* mutants. The genotypic sex (XY or XX) was determined based on the presence of the male sex-determining gene *dmy*, while the phenotypic sex (male or female) was assessed according to the secondary sex characteristics. As shown in Fig. 4A and B, wild-type XX individuals exhibit a fused dorsal fin and a triangular-shaped anal fin, along with prominent urinogenital papillae. In contrast, both XX and XY mutant fish displayed a forked dorsal fin and a parallelogram-shaped anal fin, accompanied by the loss of urinogenital papillae, which are typical secondary sex characteristics of wild-type XY individuals. These findings indicate that the XX *tatdn2* mutants undergo a female-to-male sex reversal, resulting in all-phenotypically male mutants.

We next examined the primary sex characteristics of mutant fish using the *vasa:EGFP/sox9b:DsRed* double transgenic stain. Since the gonads of mutant fish at 120 dpf were severely degenerated, gonads at 60 dpf were reexamined. As shown in Fig. 4C, gonads from both XX and XY mutants exhibit an extremely thin tube-like structure. The mutant gonads showed azoospermia, with DsRed-labeled somatic cells but no EGFP-labeled germ cells. Histological sections revealed germ cells at different stages in WT gonads, but none in mutants (Fig. 4D). The mutant gonad structure was a hollow tube consisting of a single internal epithelial layer and a thick external stromal layer, similar to previous observations of germ-cell-deficient medaka gonads [60].

We then analyzed expression of sex-specific markers [40, 61] in the mutants. Transcripts of germ cell markers (*vasa, dnd1*) were nearly undetectable in mutant gonads. Female somatic markers (*foxl2, aromatase*) showed minimal expression, while male somatic markers (*dmrt1, p45011b, cyp17, sox9b*) were detected in both XX and XY (Fig. 4E).

Overall, these results indicate that *tatdn2* null mutants are completely devoid of germ cells and undergo female-to-male sex reversal.

### Loss of *tatdn2* results in PGC depletion during mitotic proliferation

**Figure 5.**
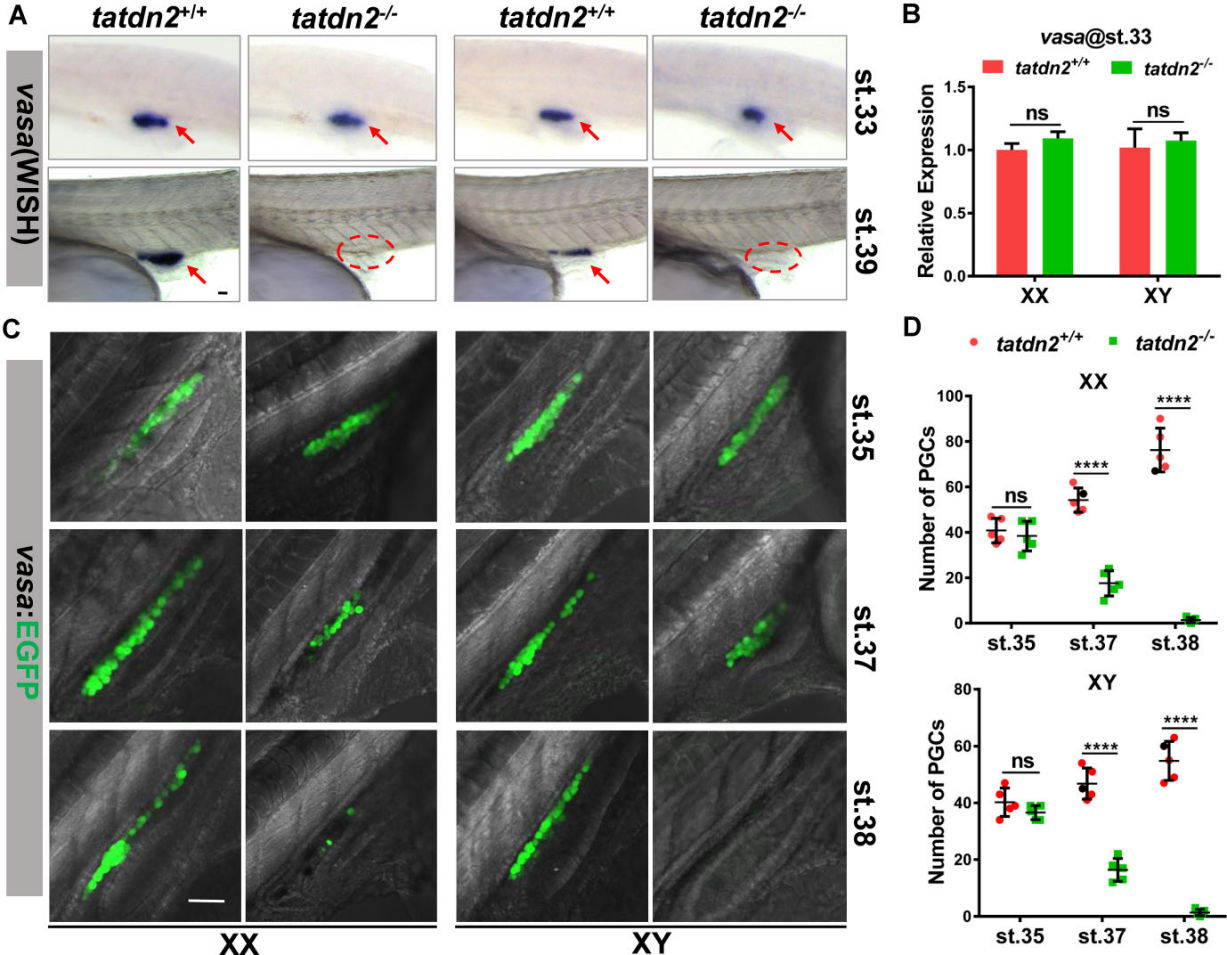
Loss of *tatdn2* results in PGC depletion during mitotic proliferation. **(A)** Expression of the PGC marker *vasa* in *tatdn2* mutants by whole mount in situ hybridization (WISH) at stages 33 and 39. The gonadal primordium is indicated by red ellipses and *vasa* transcripts are marked by red arrows. Anterior is to the left. Lateral view. Scale bar = 50 µm. **(B)** Quantification of *vasa* mRNA levels at stage 33 by qRT-PCR. **(C)** Changes in PGC numbers in *tatdn2* mutants during embryonic development. Z-stack images of live embryos were captured at designated stages. Maximum intensity projection is shown in lateral view with anterior to the top right. Scale bar = 50 µm. **(D)** Quantification of PGC numbers in XX and XY embryos. Note that PGC numbers are indistinguishable between XX and XY at stage 35, regardless of *tatdn2* genotype.

Since *tatdn2* mutants are infertile males lacking germ cells, we sought to determine the developmental stage when germ cells are eliminated. We first examined PGC development using the endogenous *vasa* marker. As teleost PGC specification depends on maternally deposited mRNAs, zygotic *tatdn2* mutation did not affect PGC specification. At stage 33, when PGCs complete migration to the gonadal primordium, both spatial distribution and abundance of *vasa* transcripts remain normal in mutants (Fig. 5A and B). However, by stage 39, when sex differentiation initiates and PGCs undergo sexually dimorphic proliferation, *vasa* mRNA becomes undetectable by WISH in mutants (Fig. 5A). qRT-PCR confirmed dramatic reduction of *vasa* and other PGC markers at this stage (Supplementary Fig. S7), indicating PGC depletion occurs between migration completion and sex differentiation onset.

We performed time-lapse imaging of *vasa:EGFP* transgenic fish to monitor PGC dynamics. At stage 35, mutant PGC (EGFP positive) numbers showed no significant difference from wild-type in both XX and XY embryos (Fig. 5C and D), consistent with normal migration observed at stage 33. This suggests initial proliferation remains unimpeded, potentially due to residual maternal *tatdn2* transcripts. However, by stage 37, PGC numbers declined dramatically in mutants, with only sporadic malformed cells detectable at stage 38. Complete absence of EGFP-labeled PGCs by stage 39 (Fig. 5C and D) aligned with endogenous *vasa* expression patterns measured by WISH and qRT-PCR (Fig. 5A and Supplementary Fig. S6), demonstrating depletion during sexually dimorphic proliferation.

These results demonstrate that *tatdn2* loss causes PGC depletion specifically during mitotic proliferation stages.

### Absence of PGCs is not caused via gonadal somatic cells in *tatdn2* mutants

**Figure 6.**
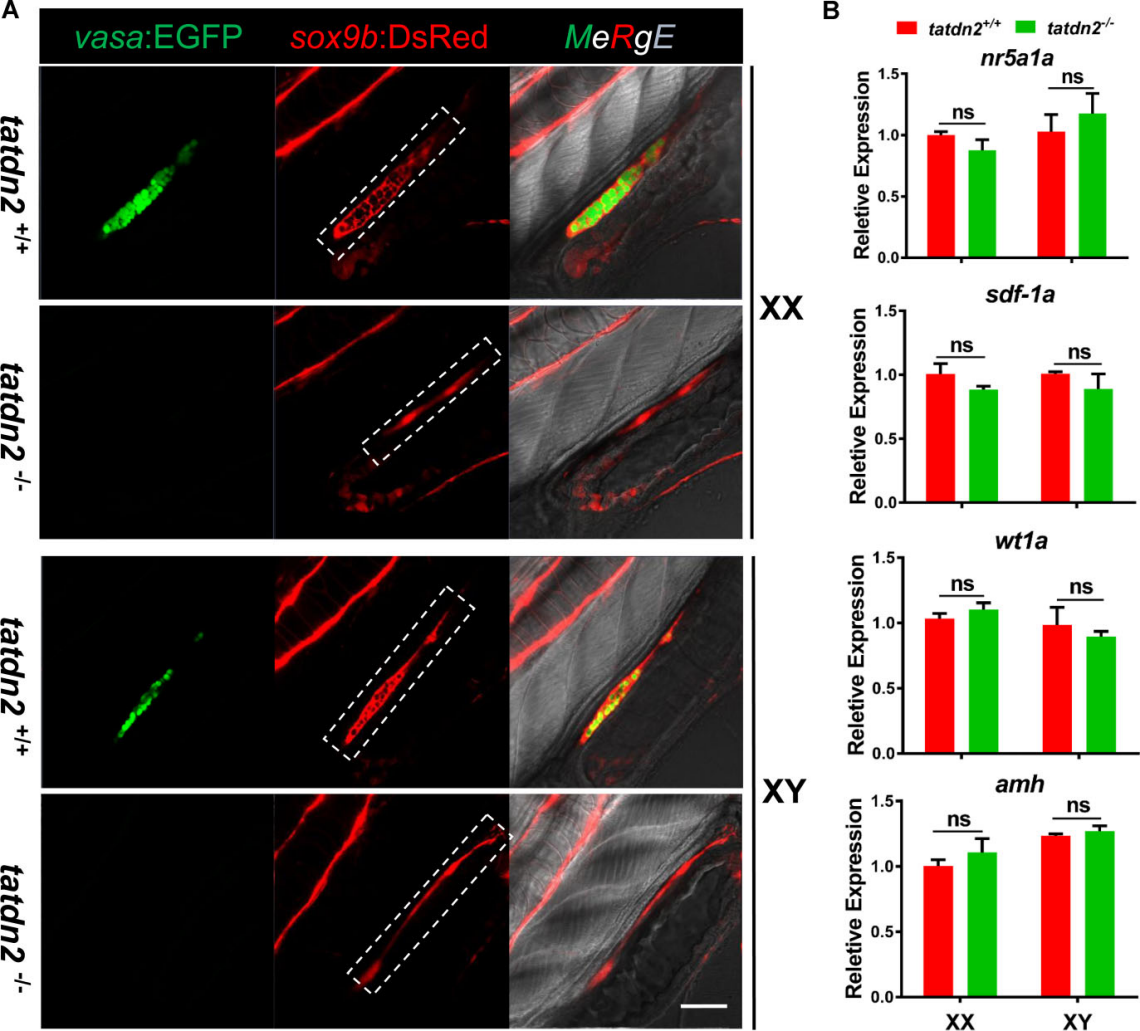
Knockout of *tatdn2* does not affect early gonadal somatic cell development. **(A)** Single confocal plane of representative gonads from live fry at stage 39. PGCs are labeled with EGFP (green), and somatic cells are labeled with DsRed (red). Lateral view with anterior to the top right. Scale bar = 50 µm. The common precursor of supporting cells, labeled with DsRed, is marked with a dashed box in the abdominal region. **(B)** qRT-PCR analysis of marker genes for gonadal somatic cell progenitors. Fry were separated into head and trunk regions, with the head used for genotyping and the trunk for qRT-PCR.

Knockout of *tatdn2* results in PGC depletion after arrival at the gonadal primordium. It is known that somatic niche plays an important role in the maintenance of PGCs [62, 63]. To ensure that developmental defects of gonadal somatic cells do not contribute to the disappearance of PGCs in *tatdn2* mutants, we examined the differentiation of gonadal somatic cells in the *vasa:EGFP/sox9b:dsRed* double transgenic fish line. According to a previous report, *sox9b* labels the common precursor of both female and male supporting cells at early stages [58]. As shown in Fig. 6, at stage 39, when sex differentiation begins, PGCs in *tatdn2* mutants have vanished, as indicated by endogenous transcripts of PGC marker gene *vasa*, together with EGFP signal driven by transgenic *vasa* promoter. On the contrary, *sox9b:dsRed* signal labeling the gonadal primordium in the abdominal region is clearly visible, indicating that the precursor of supporting cells is not affected. We also examined the expression of other gonadal somatic cell progenitor markers, including *sdf-1a, nr5a1a, amh*, and *wt1a* [64–66]. qRT-PCR results revealed that the endogenous expression of these markers remained unchanged despite a drastic reduction in the PGC marker’s expression (Supplementary Fig. S7). These results suggested that the development of gonadal soma precursor cells seems to be intact at stage 39 when PGCs are completely depleted in *tatdn2* mutants.

Furthermore, the absence of PGCs in the *tatdn2* mutant is not due to defects of gonadal somatic cells. In contrast, the female-to-male sex reversal and degeneration of adult gonads are a consequence of the deficiency of PGCs, as previously reported [40, 60]. This indicates that *tatdn2* is essential for the survival and proliferation of PGCs during development.

### *Tatdn2* deficiency causes R-loop accumulation and DNA damage-induced apoptosis

**Figure 7.**
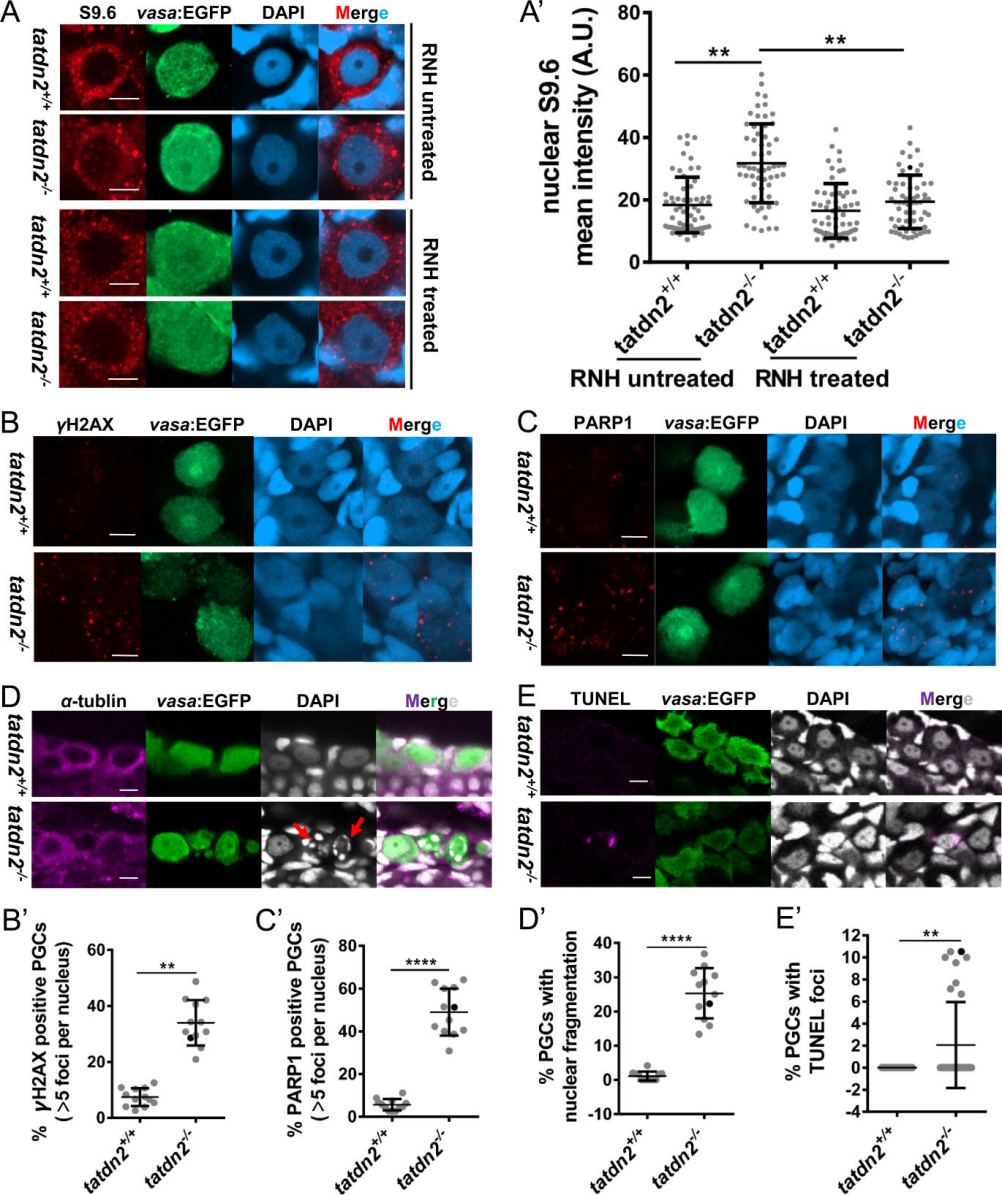
Loss of *tatdn2* leads to R-loop accumulation, DNA damage, and apoptosis in PGCs. **(A)** R-loop immunofluorescence (IF) staining using the S9.6 antibody. Embryos were treated with or without RNase H (RNH) to confirm S9.6 specificity. Nuclear fluorescence intensity of S9.6 was measured based on DAPI signal. **(B)** DNA damage IF staining with γH2AX antibody. **(C)** DNA damage IF staining with Parp1 antibody. Foci numbers of γH2AX and Parp1 per nucleus were quantified. **(D)** Nuclear staining of *tatdn2* mutant PGCs with cytoskeleton staining. Nuclei were stained with DAPI (gray), and microtubules were stained with α-tubulin antibody (purple). Fragmented nuclei are indicated by red arrows. **(E)** TUNEL staining for apoptosis detection. (**A’**–**E’**) Statistical results for corresponding experiments. All embryos were XX *vasa:EGFP* transgenic fish, with A–C at stage 36 and D and E at stage 37. Data from three independent experiments are shown. A.U., arbitrary units. Scale bar = 5 µm.

We further investigated the underlying mechanism of PGC depletion during mitotic proliferation in *tatdn2* mutants. Human TATDN2 has recently been found to function as a structure-specific RNase to resolve R-loops *in vitro* and be required for the response to replication stress in BRCA1-deficient cancer cells [36]. On the other hand, mitotic mouse PGCs have recently been characterized by high levels of TRCs and consequent R-loops [23–26]. Therefore, we first analyzed whether *tatdn2* depletion increased the accumulation of R-loops. As shown in Fig. 7A and A’, analysis of R-loops by immunostaining using the S9.6 anti-DNA–RNA hybrids antibody revealed a significant increase of the S9.6 nuclear signal in *tatdn2* knockout PGCs compared with the wild type. In contrast, S9.6 signal levels in the surrounding somatic cells were unaffected (Supplementary Fig. S8). To validate the identity of this increased nuclear signal, we performed a series of enzymatic controls. The elevated signal was resistant to co-treatment with RNase III and T1, which degrade double-stranded and single-stranded RNA, respectively. However, the signal was sensitive to RNase H, which specifically digests the RNA strand of DNA–RNA hybrids. These results confirm that the detected nuclear immunofluorescence signals represent bona fide R-loops (Supplementary Fig. S9). Therefore, the accumulation of these R-loops in *tatdn2*-deficient PGCs demonstrates that *tatdn2* participates in their resolution in proliferative PGCs.

As unresolved R-loops can lead to DNA damage response [67], we then measured the level of $\gamma$ H2AX, a well-established sensitive marker of DNA damage [68]. We found that $\gamma$ H2AX foci were significantly increased in *tatdn2*-depleted PGCs compared to normal levels (Fig. 7B and B’). We also measured the level of Parp1, a sensor of DNA damage that promotes the accumulation of downstream DDR proteins [69]. Similarly, the number of Parp1 foci was increased in *tatdn2* mutants (Fig. 7C and C’). These results indicated that DNA damage accumulated in mitotic PGCs due to the loss of *tatdn2*.

It is well established that the accumulation of DNA damage activates cell cycle checkpoints and leads to an increase of apoptosis in germline [12, 17]. We then detected signals of apoptosis in *tatdn2* mutants. Nuclear fragmentation is the most important morphological feature while cells undergo apoptosis [70] and can easily be observed by nuclear staining. Therefore, nuclear staining was performed to observe the nuclear morphology. As shown in Fig. 7D and D’, a high proportion of fragmented nuclei is observed in *tatdn2*-KO PGCs compared to that of the wild type. In addition, a TUNEL assay was performed to detect apoptosis. As shown in Fig. 7E and E’, apoptotic signals are frequently detected in PGCs of *tatdn2*-KO embryos. By contrast, we rarely observed apoptosis in germ cells of wild-type embryos. This distinct difference suggested that accumulated DNA damage in *tatdn2*-KO PGCs leads to cell apoptosis.

Altogether, these results imply that *tatdn2* is required for R-loop-associated DNA damage repair to safeguard genome stability in mitotic PGCs.

## Discussion

To date, the *in vivo* functions of TatD proteins have been studied in lower organisms such as bacteria, yeasts, protozoan parasites, and nematodes. Our understanding of their roles in higher organisms, especially vertebrates, is still limited. Recent research has identified human TATDN2 as a structure-specific RNase that participates in DNA repair under replication stress by resolving R-loops in BRCA1-deficient cancer cells [36]. Moreover, knockout of mouse *Tatdn2* by the International Mouse Phenotype Consortium resulted in embryonic lethality [71]. These observations indicate the biological relevance of *tatdn2* in physiological contexts. In this study, we investigated the biological functions of *tatdn2* using medaka fish as the model. We demonstrated that medaka *tatdn2* is specifically and continuously expressed in germ cells after specification, and knockout of *tatdn2* led to R-loop accumulation, increased DNA damage, and apoptosis in PGCs. These results suggest that *tatdn2* is involved in R-loop-associated DNA damage repair to maintain genome stability during PGC mitotic proliferation. This study advances our understanding of the physiological roles of TatD nucleases and the mechanisms of DNA repair by which PGCs cope with replication stress.

### Evolutionary conservation and divergence of *TATDN2* expression patterns

Our combined experimental and bioinformatics analyses reveal germ cell-specific expression of *tatdn2* in medaka. This finding is corroborated by Atlantic salmon data showing gonad-specific *tatdn2* expression predominantly in germ cells [72]. Transcriptomic analyses across taxa demonstrate that teleost *tatdn2* displays dual characteristics: spatial restriction to germ cells (as opposed to somatic cells) coupled with temporal persistence across mitotic, meiotic, and post-meiotic developmental stages in both gonads.

Mammalian *TATDN2* exhibits distinct expression characteristics. First, it shows broad tissue distribution, including gonadal tissues. Second, within gonads it is expressed in both somatic and germ cells. Third, testicular expression is stage-specific—present in mitotic and early meiotic germ cells but absent in post-meiotic stages across examined species.

Despite these differences, conserved features emerge: both groups show *TATDN2* expression in mitotic/early meiotic germ cells. The teleost-specific germline restriction makes these species particularly suitable for investigating *tatdn2*’s in vivo germ cell functions, as demonstrated by our medaka model revealing its essential role in mitotic PGCs.

### *Tatdn2* may function in resolution of TRC-induced R-loops and act downstream of the ATM/FA pathway in mitotic PGCs

Much less is known about the mechanisms of DNA repair for PGCs to maintain genome integrity. In the present study, we provide compelling pieces of evidence that *tatdn2* is a new player involved in R-loop-associated DNA damage repair in proliferating PGCs. Human TATDN2 functions as a structure-specific RNase to resolve R-loops and is required for the response to replication stress in BRCA1-deficient cancer cells [36]. Given that proliferating PGCs have been found to encounter high levels of endogenous replication stress induced by TRCs [23], we propose *tatdn2* resolves TRC-related R-loops to mitigate replication stress in PGCs.

We still do not know the exact molecular mechanism of *tatdn2* function in PGCs, such as how *tatdn2* is activated and recruited to the site of R-loops. As mentioned above, FA/BRCA pathways play predominant and conserved roles in the resolution of TRC-induced R-loops in rapidly proliferating PGCs among vertebrates [17, 22]. There are several pieces of evidence suggesting a link between the mechanism of *tatdn2* and the FA/BRCA pathway. First, the E3 ligase BRCA1 (FANCS), which is a key component of the downstream or functional units of the FA/BRCA pathway, acts as a pivotal mediator in DNA-damage response pathways and plays essential roles in DNA homologous recombination (HR) repair and replication fork protection and repair [73]. Particularly, BRCA1 and its functional copartner BRCA2 (FANCD1) directly interact and recruit ribonucleases or helicases, such as RNase H, Senataxin, and XRN2, to the DSBs for R-loop resolution [74–76]. Interestingly, TATDN2 was identified as an interacting protein of BRCA1 through yeast two-hybrid screening in a systematic analysis of protein–protein interactions [77]. Secondly, the protein kinase ATM, which always acts upstream of BRCA1, is one of the crucial transducers in DNA-damage response pathways and plays critical roles in HR-mediated DSB repair and response to replication stress [73, 78]. Human TATDN2 was identified as a substrate for ATM in response to radiation in pancreatic ductal adenocarcinoma by phosphoproteomic analysis [79]. These mutually independent but corroborative *in vitro* pieces of evidence strongly suggest that *tatdn2* appears to act downstream of the ATM/FA pathway in PGCs, although more detailed biochemical characterization is needed.

### Sophisticated and multiple functions of TATDN2 in germ cells and cancer cells

On the one hand, our study shows that *tatdn2* mRNA is ubiquitously expressed in multiple stages of germ cells, including mitotic, meiotic, and even temporarily arrested cell types, where replication stress is low. Given the diverse types of genome instability and the fact that DNA damage responses are remarkably diverse in germ cells at different stages [80, 81], the broad expression pattern indicates that the functions of *tatdn2* in germ cells may be quite diverse and distinct, similar to many FA proteins that play divergent roles in mitotic and meiotic germ cells [17, 82–84]. On the other hand, recent research showed that TATDN2 is essential for the survival of BRCA1-deficient cancer cells but less so for BRCA1-repleted cancer cells [36]. Our findings showed that *tatdn2* is indispensable for the survival of PGCs, which are in a BRCA1-proficient background. These observations suggest that *tatdn2* functions both downstream and parallel to BRCA1, with importance varying depending on the cell type or the functional status of BRCA1.

Alterations in DDR proteins, including *TATDN2*, are associated with the development and metastasis of various cancers [85]. Dysregulation of *TATDN2* is associated with a high rate of postoperative recurrence or subsequent metastases in cancers such as uveal melanoma, pancreatic cancer, prostate cancer, hepatocellular carcinoma, and head and neck squamous cell carcinoma [86–90]. Our discovery will contribute to a better understanding of the complex pathological roles of *tatdn2* in cancers with variable BRCA1 function status.

In short, our recent work has elucidated the biological significance of the vertebrate-conserved *tatdn2* gene at the organismal level, identifying a novel player in R-loop-related DNA damage response crucial for maintaining genome integrity in PGCs. These findings not only deepen our understanding of the physiological functions of TatD nucleases but also shed light on mechanisms underlying genome integrity maintenance during PGC mitotic proliferation. Additionally, our study offers new insights into TATDN2’s complex pathologic roles in cancer biology.

## Supplementary Material

gkaf1289_Supplemental_Files

## Data Availability

The data underlying this article are available in the article and in its online supplementary data.
