# Supplementary material for: Tatdn2 is required for DNA repair to safeguard genome stability in primordial germ cells
Source: Nucleic Acids Res. 2025 Dec 17;53(22):gkaf1289. doi: 10.1093/nar/gkaf1289 (PMC12709183; doi:10.1093/nar/gkaf1289)
Supplement: gkaf1289_Supplemental_Files [file gkaf1289_supplemental_files.zip › Supplementary_Table.docx]

Supplementary table 1. Primers used in this study.

| **Gene** | **Sequence (5' to 3')** | **Direction** | **Application** | **Ensemble ID** |
| --- | --- | --- | --- | --- |
| *tatdn2* | CTTCTACTGTCCTACTTCA | Forward | Genotyping | ENSORLG00000001485 |
|  | CTGACTGTAGAGGCAGAGC | Reverse |  |  |
| *tatdn2* | AGATCCAGCAAGTGTCGACA | Forward | Genotyping | ENSORLG00000001485 |
|  | TCCAAACTCCTCATCAGATC | Reverse |  |  |
| *dmy* | CAGAGAGTTGGATTTACGTC | Forward | Genotyping | ENSORLT000000025382 |
|  | GTCTCTAGTGCTTTAAACTAA | Reverse |  | NM_001104680 |
| *tatdn2* | CACGGTGCAGCCGCCACAC | Forward | Probe preparation | ENSORLG00000001485 |
|  | TAATACGACTCACTATAGGGACTATCCACAATCTCTCTC | Reverse |  |  |
| *vasa* | GTGCGAGTCGTTGAAGAACA | Forward | Probe preparation | ENSORLG00000020672 |
|  | TAATACGACTCACTATAGGGGTGGACGTAGTCGTCGATGG | Reverse |  |  |
| *tatdn2* | CCAAAGGCAGATACTAGAGACT | Forward | Real-time qPCR^a^ | ENSORLG00000001485 |
|  | CAGGTCGGCAGTAAGATCAGG | Reverse |  |  |
| *tatdn2* | CCAAAGTTACACCTCAGAGCC | Forward | Real-time qPCR^b^ | ENSORLG00000001485 |
|  | CAGAATCTTGACCCTGTTCGCCGTC | Reverse |  |  |
| *β-actin* | CAGGGAGAAGATGACCCAGA | Forward | Real-time qPCR | ENSORLG00015014307 |
|  | GGGTCACACCATCACCAGAG | Reverse |  |  |
| *vasa* | TCGGCATCCTGGCCAAGGCT | Forward | Real-time qPCR | ENSORLG00000020672 |
|  | TGTCCTGGAAAGAACCACCCTT | Reverse |  |  |
| *dazl* | GCAAAGG ATACGGGTTCGTGTA | Forward | Real-time qPCR | ENSORLG00000004848 |
|  | AGCCAGGCGGAATAGACCGA | Reverse |  |  |
| *dnd1* | GTGTGGAGAGTGTCGCCTTG | Forward | Real-time qPCR | ENSORLG00000025449 |
|  | CAGAACTGCTTCTTGAACTCTTC | Reverse |  |  |
| *wt1a* | GAGAAACCCCTACAACAGTCAT | Forward | Real-time qPCR | ENSORLG00000012424 |
|  | CCGTCGCACATCCTGAATGC | Reverse |  |  |
| *amh* | TGCTGCCACACTCACAGTCTGC | Forward | Real-time qPCR | ENSORLG00000002949 |
|  | GGCGTCTATCAGGTTTTGGTTCA | Reverse |  |  |
| *foxl2* | TCTCGGGTGCAGAGCATGGC | Forward | Real-time qPCR | ENSORLG00000020203 |
|  | GGTGGCCGGACTCAGTTGCT | Reverse |  |  |
| *cyp17* | CCTGGAGGTTCCACCGAAAG | Forward | Real-time qPCR | ENSORLG00000019226 |
|  | GACTGGGCCTCTGTACAGATGA | Reverse |  |  |
| *p45011b* | CTCTGGAGCCATGGGCAAC | Forward | Real-time qPCR | ENSORLG00000010480 |
|  | GATCTCCATTCTTCTCCGTTCT | Reverse |  |  |
| *aromatase* | CTGGGTGTTCCTGTTGACGAG | Forward | Real-time qPCR | ENSORLG00000002949 |
|  | GCATCTTGGAGCTCCTGGGC | Reverse |  |  |
| *sox9b* | TCCAGGAGAACATTCAGGTC | Forward | Real-time qPCR | ENSORLG00000007960 |
|  | GCCAAAGTCTATGTTGAGCTG | Reverse |  |  |
| *dmrt1a* | TCCGGCTCCACAGCGGTC | Forward | Real-time qPCR | ENSORLG00000020817 |
|  | TCCGCAATCAGCTTGCATTTGG | Reverse |  |  |
| *nr5a1* | CAGGAGGTCCAGCTGTCATC | Forward | Real-time qPCR | ENSORLG00000016486 |
|  | TCCAGCAGCTTTACATTGGG | Reverse |  |  |
| *sdf-1a* | TATGCGCCTCAAACACACGCA | Forward | Real-time qPCR | ENSORLG00000027241 |
|  | CCTGTTTGTCTTCAGTTTAGCAATC | Reverse |  |  |
| *piwi1* | CTACAACATCCTCTTCAAAAGG | Forward | Real-time qPCR | ENSORLG00000013695 |
|  | GCCTGGCCAGATCGTTAGGT | Reverse |  |  |

The sequence of T7 promoter for in vitro transcription is underlined.

a. This primer set was used for detection of tatdn2 transcripts in WT fish.

b. This primer set was used for detection of tatdn2 transcripts in tatdn2 mutants.
