# Supplementary material for: Tatdn2 is required for DNA repair to safeguard genome stability in primordial germ cells
Source: Nucleic Acids Res. 2025 Dec 17;53(22):gkaf1289. doi: 10.1093/nar/gkaf1289 (PMC12709183; doi:10.1093/nar/gkaf1289)
Supplement: gkaf1289_Supplemental_Files [file gkaf1289_supplemental_files.zip › tatdn2_Supplemental_Figures.pdf]

Tatdn2 is required for DNA repair to safeguard genome stability in primordial germ cells

Supplementary Figures

Hanqiao Shang[1], Puxuan Jiang[1,2], Zehui Wang[1,2], Zhaojun Shan[1,2], Yuze Chen[1,2], Ting Zhang[1], Yingshu Li[1], and Qiang Tu[1,2,*]

[1]*State Key Laboratory of Molecular Developmental Biology, Institute of Genetics and Developmental Biology, Chinese Academy of Sciences, Beijing 100101, China*

[2]*University of Chinese Academy of Sciences, Beijing 100049, China*

[*]*Corresponding authors: qtu@genetics.ac.cn*

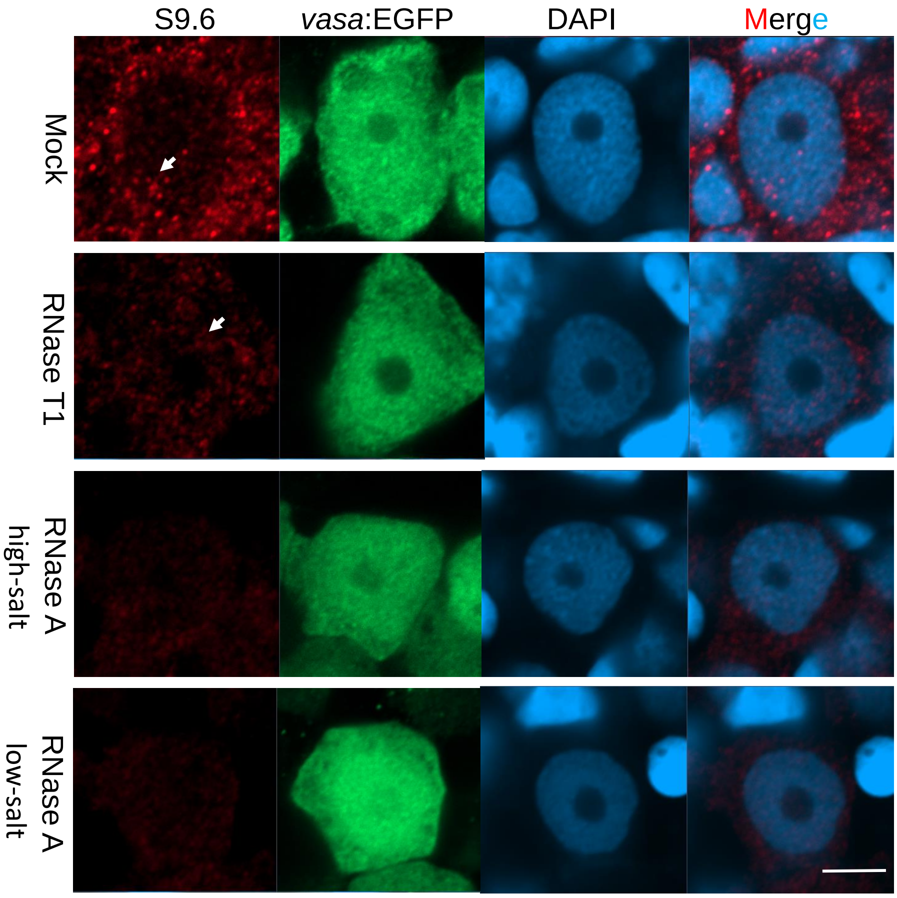

Supplementary Figure 1: RNase A retains the capacity to degrade DNA–RNA hybrids under high-salt condition (500 mM NaCl) in embryonic PGCs. Mutant embryos were used here as their S9.6 IF signals are easier to observe. Treatment conditions were based on previous reports and product manuals. Fixed embryos were treated at 37 °C for 2 h with RNase T1 (ssRNA-specific, positive control; EN0541, ThermoFisher, 5 U/µL) or RNase A under high-salt (test; EN0531, ThermoFisher, 0.1 µg/µL) and low-salt (negative control) conditions. In the high-salt RNase A group, no typical S9.6 nuclear foci were observed, similar to the negative control group. This indicates that RNase A failed to confine its substrate specificity to ssRNA and retained the capacity to degrade DNA–RNA hybrids. White arrows indicate typical nuclear foci in the mock and RNase III & T1 groups. All data were obtained under the same IF and imaging parameters. Scale bar = 5 µm.

A

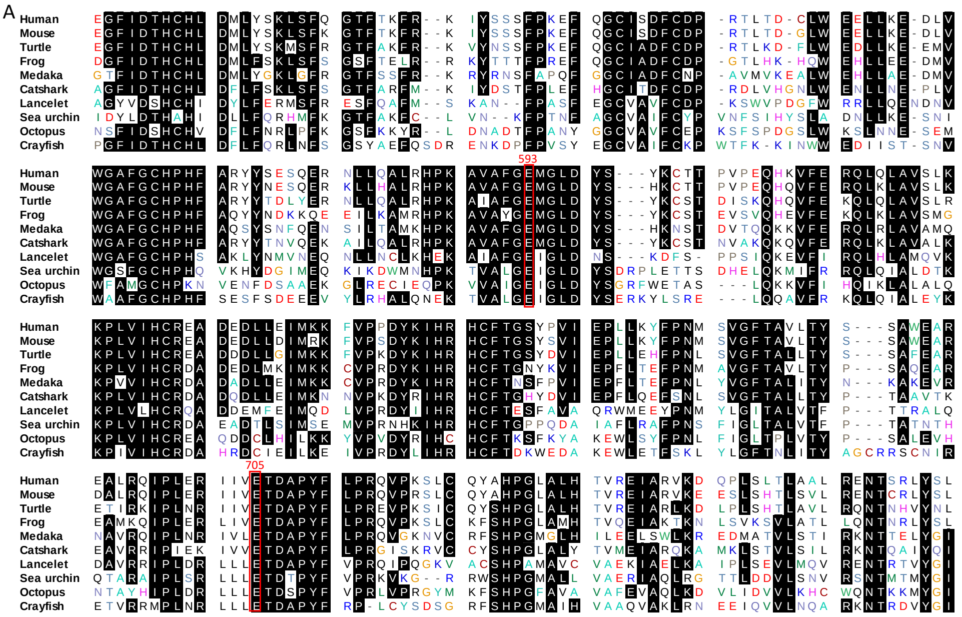

B

	Interspecies TATDN2s (/Human)										TATDN1*
	Vertebrata						Cephalochordata	Echinodermata	Lophotrochozoa	Ecdysozoa	E.coli /Human
	Mouse	Turtle	Frog	Medaka	Zebrafish	Shark	Lancelet	Sea urchin	Octopus	Crayfish	
**Identity**	92.19%	81.78%	72.53%	63.50%	63.14%	69.34%	49.64%	44.52%	43.66%	42.27%	32.52%
**Similarity**	96.65%	94.05%	87.91%	81.02%	82.12%	81.75%	71.07%	65.02%	66.55%	64.26%	48.47%

Supplementary Figure 2: Sequence conservation of the TatD DNase domain of TATDN2 proteins from coelomates. (A) Multiple sequence alignment of the TatD DNase domain of TATDN2 from selected species of both vertebrates and invertebrates using ClustalW algorithm. The two amino acid residues E593 and E705 essential for the catalytic activity of human TATDN2 (in red box) are conserved in TATDN2 across coelomates. (B) Sequence identity and similarity of the TatD DNase domain of TATDN2 between human and other species. The identity and similarity between human TATDN1 (NP_114415) and *Escherichia coli* TatD (YP_026271) was used as a control. Vertebrates: human (*Homo sapiens*) NP_055575, mouse (*Mus musculus*) NP_001028635, turtle (*Terrapene triunguis*) XP_024063304, frog (*Xenopus tropicalis*) XP_031757020, zebrafish (*Danio rerio*) NP_001122165, Japanese medaka (*Oryzias latipes*) XP_011472966, catshark (*Scyliorhinus canicula*) XP_038666707. Invertebrates: lancelet (*Branchiostoma floridae*) XP_035663790, purple sea urchin (*Strongylocentrotus purpuratus*) XP_030828787, octopus (*Octopus bimaculoides*) XP_014771063, crayfish (*Cherax quadricarinatus*) XP_069952795.

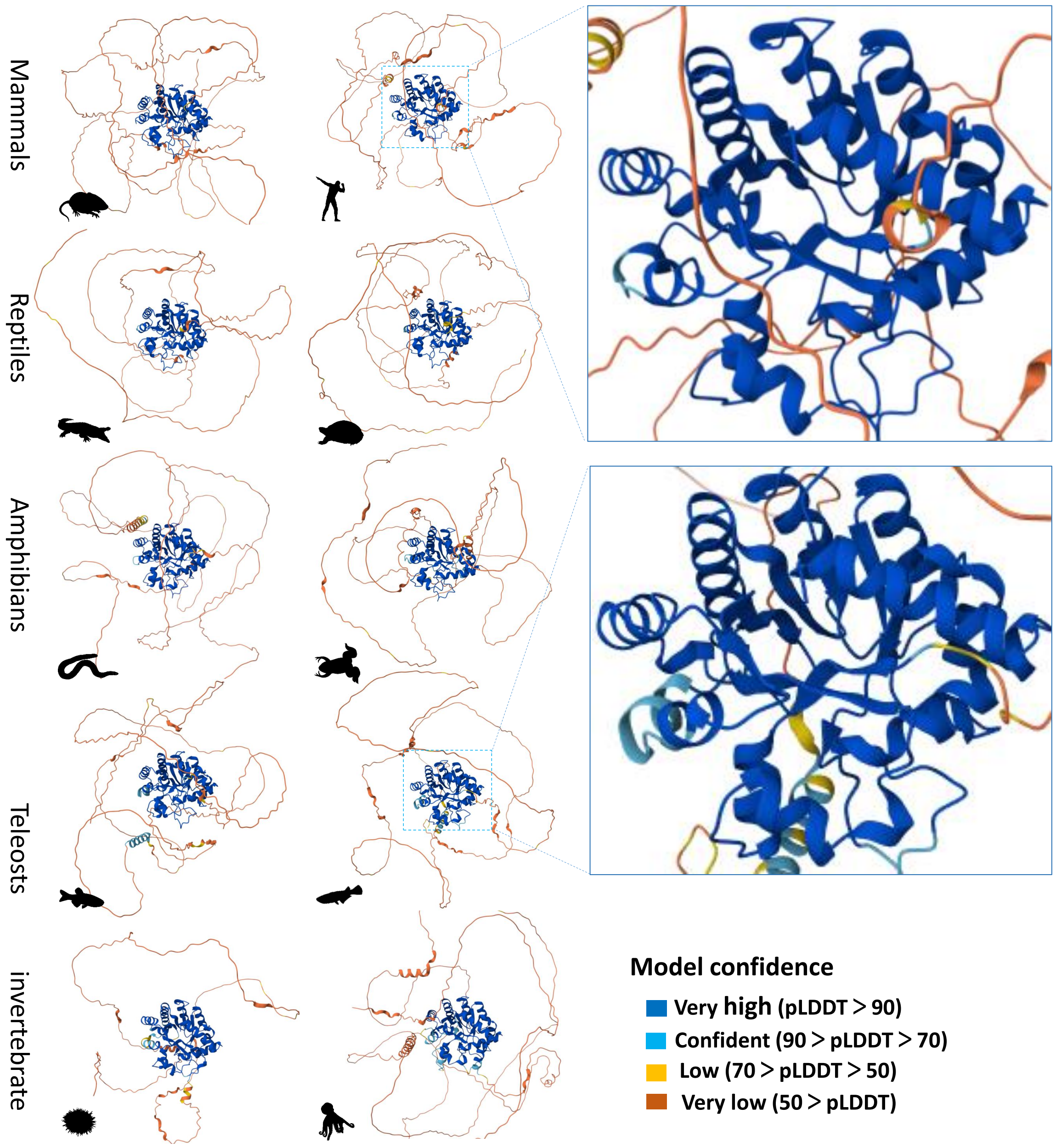

Supplementary Figure 3: 3D structural similarity of TATDN2 amongst major vertebrate and invertebrate groups. The predicted structures of full-length TATDN2 were selected from AlphaFold3 with model confidence color-coded. Some regions with low pLDDT may be unstructured in isolation. The C-terminus containing the TatD DNase domain shows structural similarity to human TATDN2, suggesting conserved biochemical functions. UniProtKB accessions: human (*Homo sapiens*) (Q93075), mouse (*Mus musculus*) (B7ZNL9), crocodile (*Crocodylus porosus*) (A0A7M4F176), turtle (*Terrapene triunguis*) (A0A674K1K8), caecilian (*Microcaecilia unicolor*) (A0A6P7YH98), frog (*Xenopus tropicalis*) (A0A803JPW3), medaka (*Oryzias latipes*) (H2L7L9), zebrafish (*Danio rerio*) (F1QY16), purple sea urchin (*Strongylocentrotus purpuratus*) (A0A7M7N1K6), octopus (*Octopus bimaculoides*) (A0A0L8GWM6). Note: Purple sea urchin TATDN2 prediction is N-terminal truncated due to incomplete gene annotation.

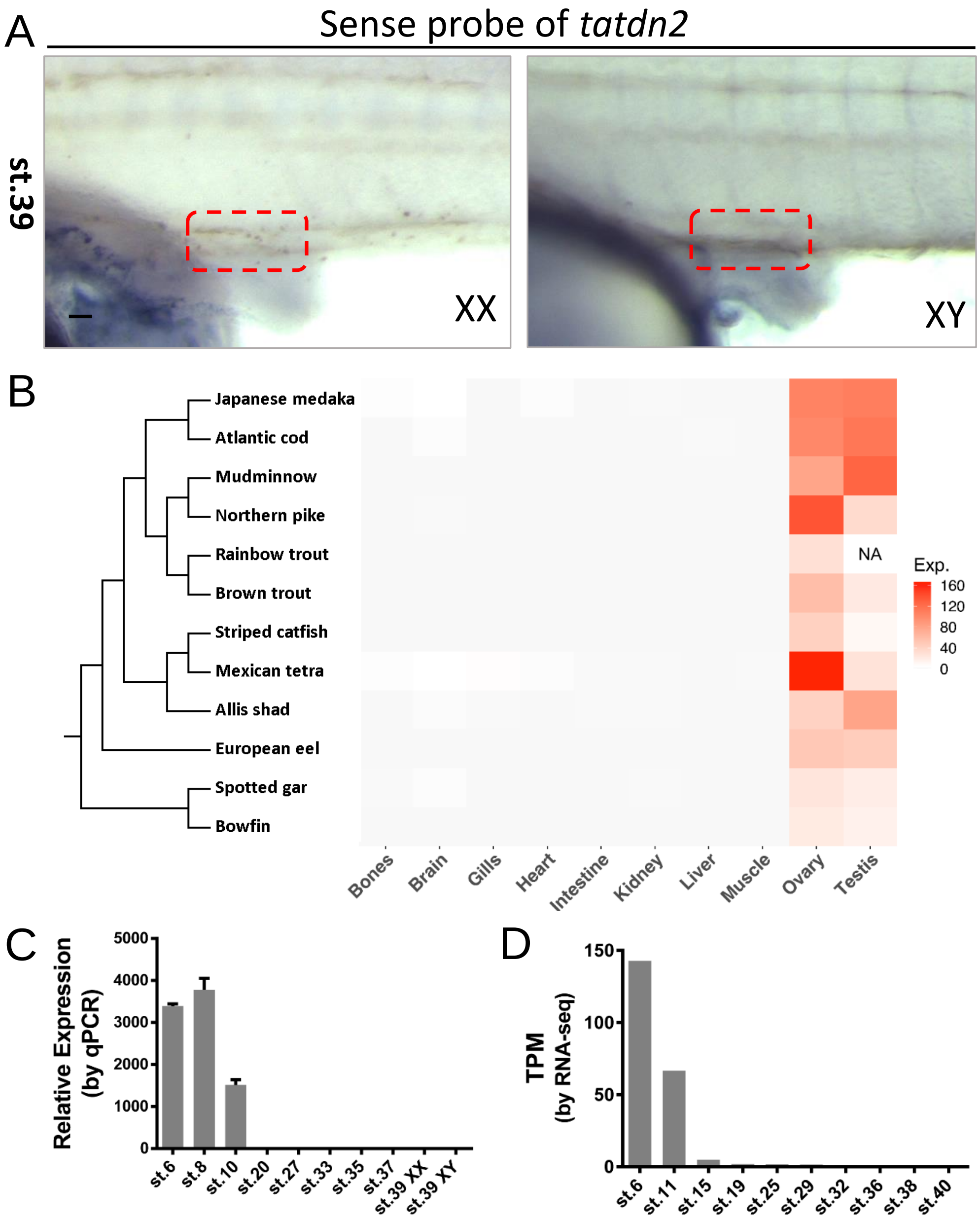

Supplementary Figure 4: Expression pattern of *tatdn2* during medaka embryonic development and in adult tissues of other teleosts. (A) WISH using sense probe of *tatdn2* (negative control). Gonadal primordium indicated by red boxes. Scale bar = 20 µm. (B) Tissue distribution in teleosts. The RNA-seq data was retrieved from FEVER database (https://fever.sk8.inrae.fr/). Data from 12 species shows conserved gonad-specific expression. (C) qRT-PCR and (D) RNA-seq analysis of *tatdn2* expression during embryogenesis. The RNA-seq data comes from a previous report of our lab (http://tulab.genetics.ac.cn/medaka_omics/).

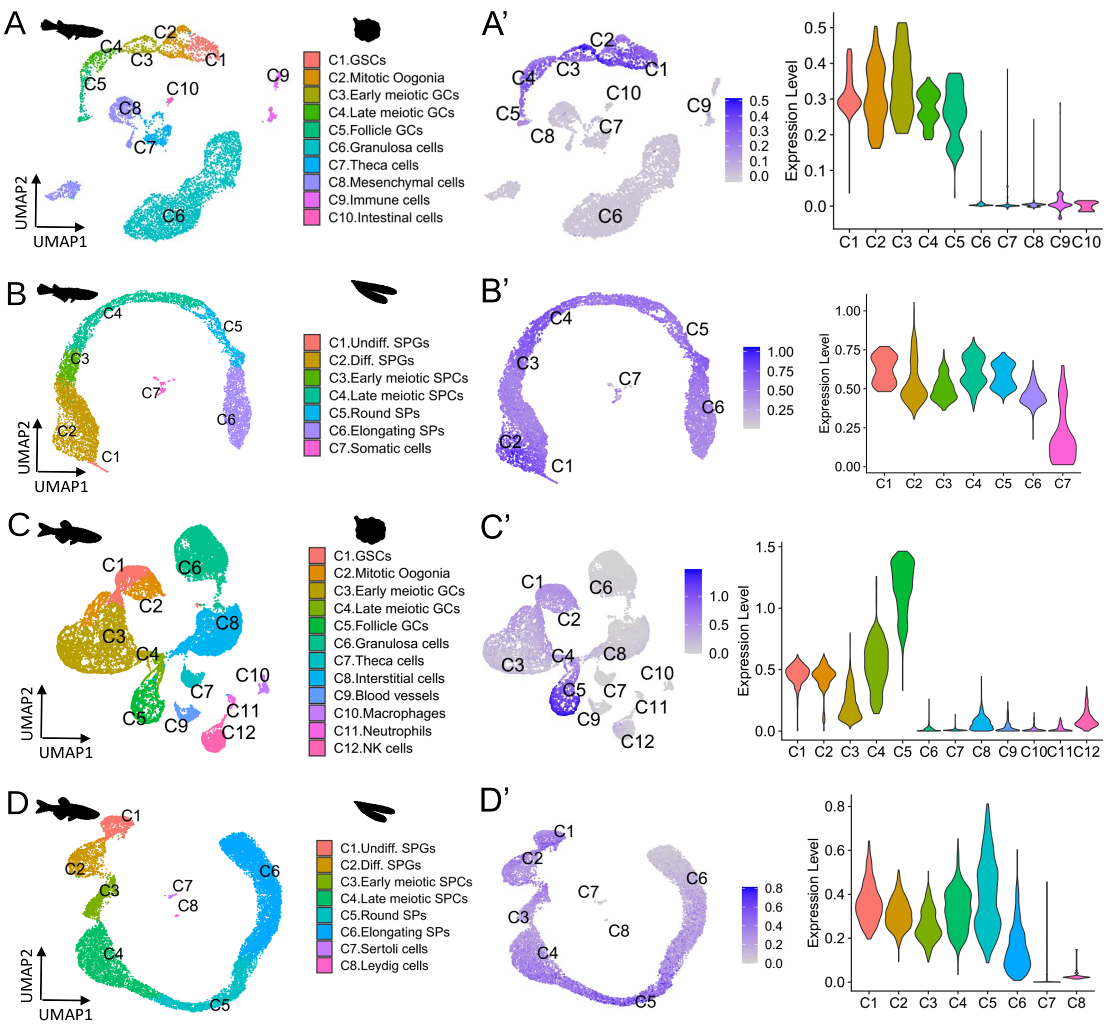

Supplementary Figure 5: Tatdn2 expression in differentiated gonads of medaka (A, B) and zebrafish (C, D). ScRNA-seq datasets of medaka were generated by our lab, and will be published elsewhere. ScRNA-seq datasets of zebrafish were downloaded from public database. (A-D) UMAP visualization of cell clusters in ovaries and testes. (A'-D') Tatdn2 expression shown in UMAP plots (right) and violin plots (left). Abbreviations: GSCs (Germline stem cells), SPGs (Spermatogonia), SPCs (Spermatocytes), SPs (Spermatids), GCs (Germ cells).

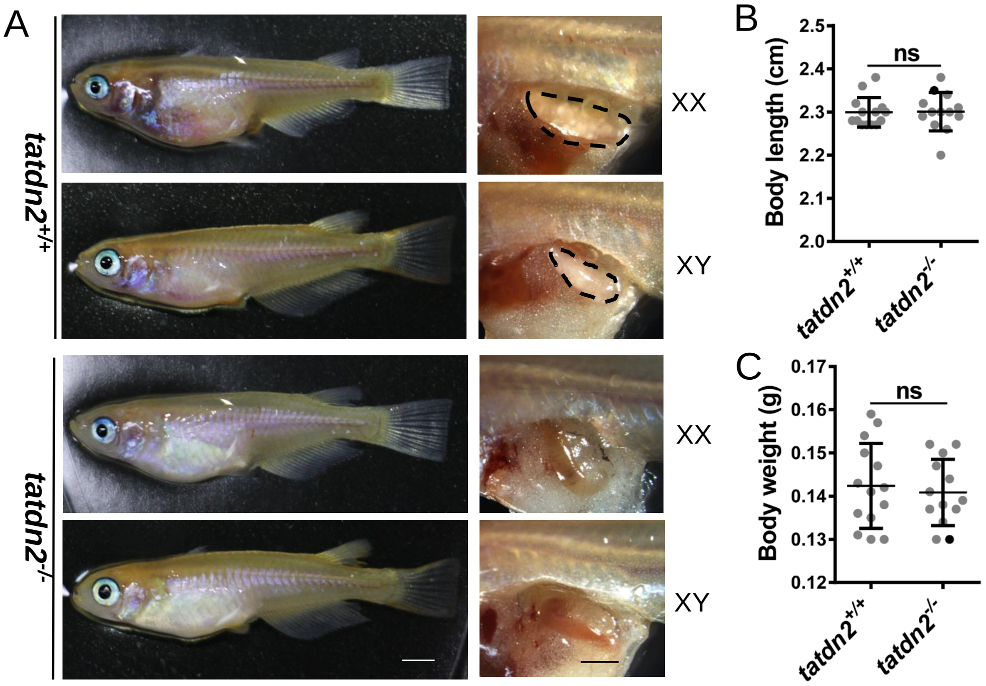

Supplementary Figure 6: Morphology and growth of *tatdn2* mutant adults. (A) Adult morphology (120 dpf) and gonad dissection. Left panel: overall morphology of representative adult. Right panel: anatomical view of dissected trunks with the gonad region. Ovary and testis are outlined with black dotted boundary. Gonads of mutant fish at about 120 dpf are too thin to be observed due to serious degeneration. Scale bar = 2 mm. Body length (B) and body weight (C) of *tatdn2* mutant adults. All fish are at about 120 dpf. Since *tatdn2* mutants are all phenotypically male, only WT males are served as control.

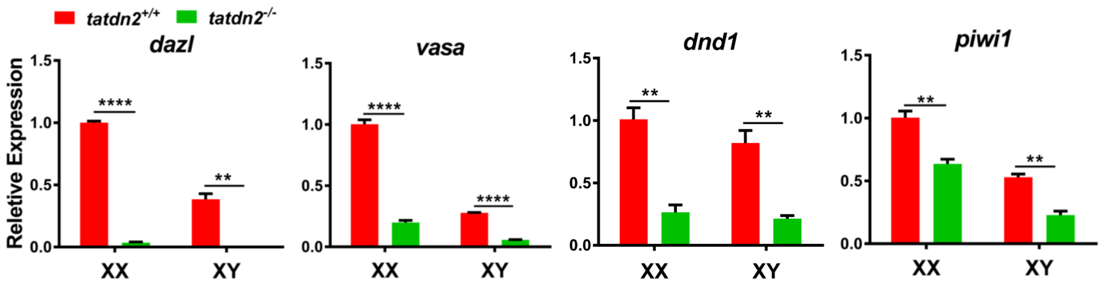

Supplementary Figure 7: Gene expression of marker genes of PGCs. qRT-PCR analysis of endogenous expression of marker genes including *vasa*, *dazl*, *dnd1* and *piwi1*. Fry were separated into head and trunk regions. The head part was used for genotyping, while the trunk region containing gonad was used for qRT-PCR detection.

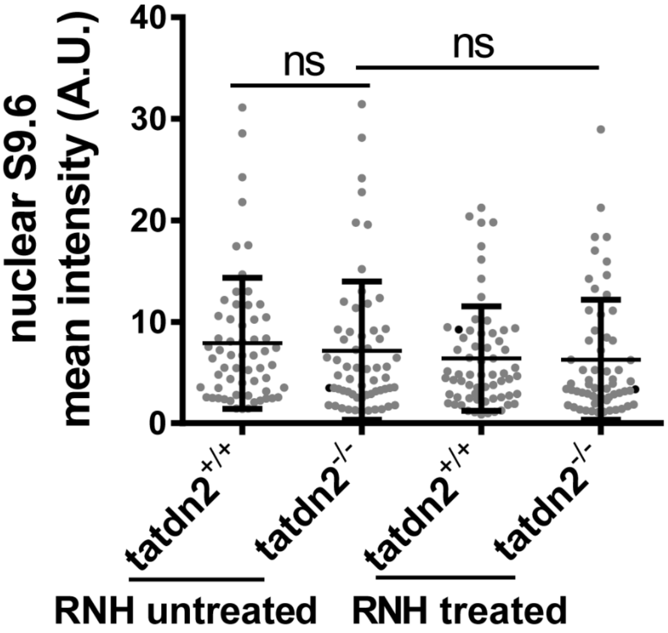

Supplementary Figure 8: The level of S9.6 signals in somatic cells. Experimental details were given in Fig. 8. Statistical results show the level of S9.6 signals in somatic cells surrounding PGCs was not affected in mutant and treatment groups.

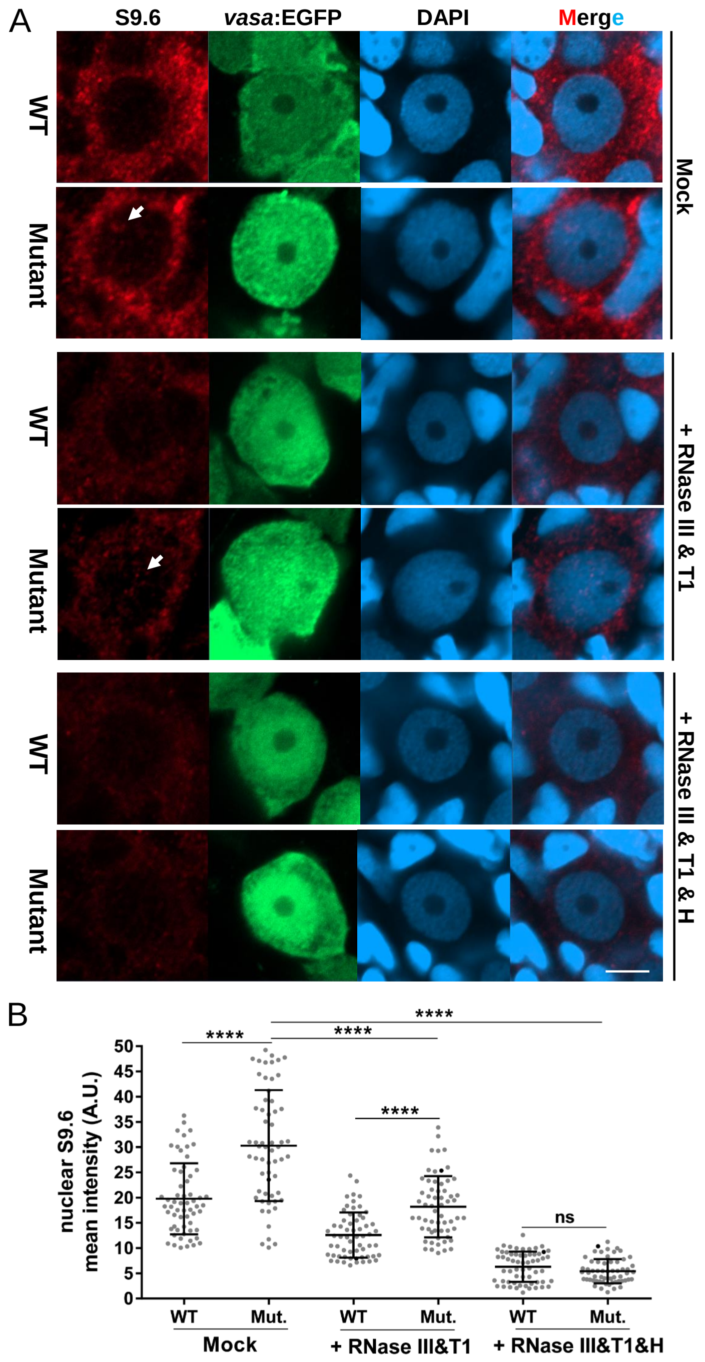

Supplementary Figure 9: Accumulation of R-loops in *tatdn2* mutants is resistant to RNase III& T1 co-treatment, but sensitive to RNase H treatment. Fixed embryos of WT or *tatdn2* mutants at stage 36 were untreated (Mock) or treated by RNase H, RNase III &T1, RNase III&T1&H separately at 37 °C for 2 h. RNase III (M0245S, NEB, 10 U/mL), RNase T1 (EN0541, ThermoFisher, 5 U/μL), RNase H (M0297S, NEB, 100 U/mL). Enzymatic treatments were done in staining buffer supplemented with 3 mM $MgCl_2$. The results for the RNase H-treated group, which were similar to those in Fig 7A of our manuscript, are not shown here to save space. White arrows indicate the typical nuclear foci by S9.6 staining in mock and RNase III & T1 group (the positive control). All images were acquired under the same conditions and imaging parameters. Scale bar = 5 μm.
